# From Oxidative Stress to Fibrotic Remodeling: Integrating Redox Biology, Galectin-3, and Imaging Phenotypes in Heart Failure

**DOI:** 10.3390/antiox15080919

**Published:** 2026-07-24

**Authors:** Samuel Ardelean, Andrada Ardelean, Diana-Evelyne Buzzi, Andrei-Catalin Zavragiu, Daniel Rus, Elena-Larisa Zimbru, Vlad Ioan Morariu, Ruxandra Maria Christodorescu, Adrian Sturza, Minodora Andor

**Affiliations:** 1 Doctoral School of Medicine, “Victor Babeș” University of Medicine and Pharmacy, Eftimie Murgu Square, No. 2, 300041 Timișoara, Romania; samuel.ardelean@umft.ro (S.A.); diana.mailat@umft.ro (D.-E.B.); andrei.zavragiu@umft.ro (A.-C.Z.); daniel.rus@umft.ro (D.R.); elena.zimbru@umft.ro (E.-L.Z.); 2 Department of Cardiology, Institute of Cardiovascular Diseases, Gheorghe Adam St., No. 13A, 300310 Timișoara, Romania; 3 Department V—Internal Medicine 1, “Victor Babeș” University of Medicine and Pharmacy, Eftimie Murgu Square, No. 2, 300041 Timișoara, Romania; morariu.vlad@umft.ro (V.I.M.); christodorescu.ruxandra@umft.ro (R.M.C.); andor.minodora@umft.ro (M.A.); 4 Multidisciplinary Heart Research Center, Faculty of Medicine, “Victor Babeș” University of Medicine and Pharmacy, Eftimie Murgu Square, No. 2, 300041 Timișoara, Romania; 5 Department of Rheumatology- Physical Medicine and Rehabilitation Clinic- Municipal Emergency Clinical Hospital, 300020 Timișoara, Romania; andrada.ardelean@rezident.umft.ro; 6 Center of Immuno-Physiology and Biotechnologies, Department of Functional Sciences, “Victor Babeș” University of Medicine and Pharmacy, 300041 Timișoara, Romania; 7 Center for Gene and Cellular Therapies in the Treatment of Cancer—OncoGen, Timiș County Emergency Clinical Hospital “Pius Brînzeu”, 300723 Timișoara, Romania; 8 Research Center, Institute of Cardiovascular Diseases Timișoara, Gheorghe Adam St., No. 13A, 300310 Timișoara, Romania; 9 Department of Functional Sciences-Pathophysiology, Center for Translational Research and Systems Medicine, “Victor Babeș” University of Medicine and Pharmacy, Eftimie Murgu Square, No. 2, 300041 Timișoara, Romania

**Keywords:** heart failure, oxidative stress, redox biomarkers, Galectin-3, cardiac remodeling, fibrosis, ferroptosis, HFpEF, SGLT2 inhibitors, cardiac magnetic resonance

## Abstract

Oxidative stress contributes to heart failure (HF) progression by mechanisms that go beyond hemodynamic overload, including mitochondrial dysfunction, endothelial injury, inflammation, and fibrotic remodeling. This review evaluates the relationship between redox imbalance, Galectin-3 (Gal-3), fibrosis, and imaging findings in HF. Reactive oxygen species (ROS) generated by mitochondria, nicotinamide adenine dinucleotide phosphate (NADPH) oxidases, and xanthine oxidase may disturb calcium handling, impair mitochondrial function, activate fibroblasts, and promote ferroptosis. Biomarkers of oxidative injury and antioxidant reserve, including malondialdehyde (MDA), 8-hydroxy-2′-deoxyguanosine (8-OHdG), and circulating thiols, provide information complementary to natriuretic peptides. Experimental evidence supports a context-dependent role of Gal-3 in fibro-inflammatory remodeling, whereas circulating Gal-3 should be regarded as a complementary biomarker rather than as a direct measure of myocardial fibrosis. Echocardiography assesses functional remodeling through diastolic indices, myocardial deformation, and right ventricular–pulmonary arterial (RV–PA) coupling, while cardiac magnetic resonance characterizes focal scar and diffuse interstitial remodeling using late gadolinium enhancement, native T1 mapping, and extracellular volume fraction. Therapeutic strategies are increasingly shifting from nonspecific antioxidant supplementation toward targeting ROS sources and downstream pathways, with SGLT2 inhibitors emerging as clinically relevant agents with indirect redox-modulating effects. Integrated redox, fibro-inflammatory, hemodynamic, and imaging phenotyping may refine risk stratification, although prospective validation is required before routine implementation.

## 1. Introduction

### 1.1. The Multimarker Concept in Heart Failure: Beyond Hemodynamic Stress

N-terminal pro-B-type natriuretic peptide (NT-proBNP) remains the reference biomarker for assessing myocardial wall stress and hemodynamic overload in heart failure. It has established value for diagnosis, severity assessment, and prognostic stratification. However, its interpretation may be influenced by age, renal function, body mass index, and atrial fibrillation; moreover, it does not directly capture inflammatory activation, oxidative injury, fibro-inflammatory remodeling, or cardiomyocyte injury [1].

Heart failure is a biologically heterogeneous syndrome in which hemodynamic, inflammatory, oxidative, fibrotic, metabolic, and cellular injury pathways interact over time. Accordingly, natriuretic peptides should be considered central but biologically incomplete markers. Multimarker strategies integrating non-natriuretic biomarkers may provide complementary information on inflammation, oxidative stress, myocardial injury, fibrosis, and extracardiac organ dysfunction, and may therefore improve disease phenotyping and risk stratification [2,3,4].

High-sensitivity cardiac troponins illustrate this idea by reflecting cardiomyocyte injury that may occur not only during acute ischemia, but also in the context of chronic myocardial stress, inflammation, and adverse remodeling. Thus, the objective of a multimarker approach is not to replace NT-proBNP, but to complement hemodynamic assessment with biomarkers indicating the biological mechanisms that sustain progression and remodeling in heart failure [3].

### 1.2. Oxidative Stress and Inflammation: The Mechanistic Foundation of Disease Progression

At the molecular level, heart failure progression cannot be explained solely by hemodynamic overload, but must be understood as the result of complex cellular and subcellular mechanisms in which oxidative stress plays a central role. Tsutsui et al. [5] show that excessive production of reactive oxygen species (ROS), mainly derived from mitochondria, NADPH oxidases, and xanthine oxidase, activates signaling pathways involved in myocyte hypertrophy, interstitial fibrosis, myocardial stiffness, and both systolic and diastolic dysfunction.

D’Oria et al. [6] emphasize that reactive oxygen species (ROS) are not exclusively harmful, as they also participate in physiological cellular signaling at low concentrations. However, when ROS production exceeds endogenous antioxidant capacity, redox imbalance promotes adverse cardiac remodeling. Similarly, Martins et al. [7] describe oxidative stress as a critical modulator of cardiac remodeling, linking neurohormonal activation, inflammation, calcium homeostasis disruption, matrix metalloproteinase activation, fibroblast proliferation, and extracellular matrix remodeling.

Beyond cardiomyocytes, oxidative stress also contributes to endothelial dysfunction. Dubois-Deruy et al. [8] highlight the role of endothelial nitric oxide synthase (eNOS) uncoupling, through which endothelial nitric oxide synthase becomes an additional source of superoxide rather than supporting nitric oxide-mediated vasodilation. Shaito et al. [9] and Scioli et al. [10] further show that ROS reduce nitric oxide bioavailability and promote a pro-inflammatory, vasoconstrictive, pro-thrombotic, and proliferative endothelial phenotype, consequently contributing to vascular remodeling, increased afterload, and worsening ventricular dysfunction [11].

Experimental high-fructose models additionally support the interaction between metabolic inflammation, endothelial dysfunction, and vascular remodeling, showing that rosuvastatin improved vascular reactivity and attenuated inflammatory and structural vascular changes; however, direct extrapolation to heart failure remains limited [12]. Broader inflammatory frameworks also suggest that microbiota-related immune dysregulation may contribute to endothelial injury and systemic vascular inflammation across chronic cardiometabolic diseases; however, its specific relevance to heart failure requires dedicated investigation [13].

The transition from compensated remodeling to overt heart failure is also influenced by progressive oxidative stress accumulation. Shah et al. [14] suggest that ROS excess initiates early subcellular remodeling processes, including impaired calcium handling, metalloprotease activation, apoptosis, and fibrosis. Zhang and Guo [15] reinforce this concept, describing oxidative stress as a central driver of inflammation, apoptosis, fibrosis, metabolic imbalance, mitochondrial dysfunction, and chronic cardiac remodeling.

This evidence indicates that oxidative stress and inflammation are not simply secondary consequences of heart failure, but essential mechanistic drivers of disease progression. They connect cardiomyocyte dysfunction, endothelial impairment, interstitial fibrosis, and adverse remodeling, supporting the need to integrate oxidative and inflammatory biomarkers into a broader biological assessment of heart failure.

### 1.3. Development of the Multimarker Approach

As heart failure is increasingly understood as the result of interacting hemodynamic, inflammatory, oxidative, fibrotic, myocardial injury, and organ dysfunction pathways, the need for multimarker assessment has become evident [16,17]. Szczurek and Szyguła-Jurkiewicz [18] emphasize that combining oxidative and inflammatory biomarkers, such as myeloperoxidase, with natriuretic peptides may better reflect heart failure pathophysiology and improve mortality risk stratification.

This concept is supported by Meijers et al. [3], who formulate the “beyond natriuretic peptides” paradigm and argue that natriuretic peptides alone cannot capture the full biological complexity of heart failure. Similarly, Topf et al. [4] propose organizing heart failure around major pathophysiological domains, including inflammation, fibrosis, oxidative stress, myocardial injury, and organ dysfunction. Lubrano et al. [19] further suggest that combining biomarkers derived from weakly correlated and independent pathogenic pathways may improve diagnostic and prognostic discrimination, although clinical validation is still necessary.

Pál et al. [20] also point out the potential value of emerging biomarkers related to inflammation, fibrosis, oxidative stress, endothelial dysfunction, and organ dysfunction, while noting that their routine clinical use still requires stronger evidence and standardization. Clinical studies and recent reviews support this approach. Gürgöze et al. [21] showed that a multimarker strategy in acute heart failure, using growth differentiation factor 15 (GDF-15), NT-proBNP, and troponin I, improved prognostic stratification by integrating complementary biological information. Alzaabi et al. [22] similarly argue that oxidative stress biomarkers such as malondialdehyde and free thiols may add prognostic value beyond conventional assessment.

However, Radovanovic et al. [23] show that oxidative stress biomarkers appear more useful for assessing disease severity in advanced symptomatic heart failure rather than for early diagnosis, reinforcing their complementary rather than substitutive role. Overall, NT-proBNP remains the central biomarker of hemodynamic stress, but its integration with biomarkers of inflammation, fibrosis, oxidative stress, myocardial injury, and organ dysfunction may allow deeper disease phenotyping, improved risk stratification, and future personalization of therapy.

Additional evidence suggests that combining hemodynamic and redox-related markers may improve risk assessment. Concomitant elevation of uric acid and NT-proBNP has been associated with poorer short-term outcomes in acute heart failure, while recent reviews and meta-analyses highlight the complementary prognostic and phenotyping potential of oxidative stress biomarkers, including MDA and 8-OHdG [24,25,26,27,28].

The main classes of biomarkers involved in heart failure and their clinical roles are summarized in Table 1.

### 1.4. Clinical Evidence Linking NT-proBNP to Oxidative Stress

Although NT-proBNP remains the reference biomarker in heart failure, clinical evidence increasingly suggests that it does not entirely capture the oxidative and inflammatory mechanisms involved in disease progression. Wróbel-Nowicka et al. [40] emphasize that oxidative stress and inflammation contribute to myocardial injury and cardiac remodeling beyond hemodynamic stress, supporting the integration of redox biomarkers into multimarker assessment.

Several studies support this concept. Smyła-Gruca et al. [30] showed that lower coronary sinus levels of ceruloplasmin and catalase, together with higher creatinine, were independently associated with one-year mortality in patients with advanced heart failure. Lazar-Poloczek et al. [31] further demonstrated that ceruloplasmin correlates with disease severity, New York Heart Association (NYHA) class, exercise limitation, NT-proBNP, total oxidant status (TOS), and MDA, suggesting that it reflects a relevant redox component complementary to natriuretic peptides.

The A Systems BIOlogy Study to TAilored Treatment in Chronic Heart Failure (BIOSTAT-CHF) analysis by de Koning et al. [32] also supports the link between hemodynamic stress and systemic oxidative imbalance, showing that higher NT-proBNP values and more advanced NYHA class are associated with lower serum free thiol levels, while thiol depletion predicts mortality independently of natriuretic peptides. Abdulle et al. [41] confirmed the broader cardiovascular relevance of free thiols, showing their independent association with cardiovascular events and all-cause mortality in the general population.

Multimarker models further reinforce this approach. Gtif et al. [29] integrated NT-proBNP with redox markers such as total antioxidant capacity and uric acid in a prognostic model for post-discharge mortality, while Park et al. [24] showed that patients with concomitantly elevated uric acid and NT-proBNP had the worst short-term prognosis in acute heart failure.

Recent reviews by Ng et al. [25] and Panda et al. [42] support an integrative view in which redox biomarkers, including malondialdehyde, advanced glycation end products, free thiols, and NADPH oxidase-related markers, may complement conventional cardiovascular biomarkers. NT-proBNP and oxidative stress biomarkers should not be considered competing tools, but complementary markers reflecting different biological dimensions of heart failure: hemodynamic overload on one side, and oxidative injury, inflammation, antioxidant imbalance, and remodeling severity on the other.

### 1.5. Galectin-3 at the Interface of Oxidative Stress, Inflammation, and Fibrosis

Galectin-3 is a biomarker that reflects biological processes insufficiently captured by standard hemodynamic assessment, particularly inflammation, oxidative stress, fibrosis, and structural remodeling. Lubrano and Balzan [43] describe Galectin-3 as an intermediary between oxidative stress, inflammation, and fibrosis, while Blanda et al. [35] emphasize its production by activated macrophages and its role in fibroblast activation, collagen synthesis, extracellular matrix accumulation, and pathological cardiac remodeling.

From a clinical perspective, Zaborska and Sygitowicz [36] place Galectin-3 within a multimarker paradigm, highlighting that it provides information distinct from NT-proBNP and may improve risk stratification when used together with established biomarkers. Prognostic evidence supports this role: Cheng et al. [37] showed that elevated Galectin-3 levels are associated with higher all-cause and cardiovascular mortality in chronic heart failure.

However, Galectin-3 should be interpreted cautiously and in combination with other biomarkers. Sulaiman et al. [44] showed that although elevated Galectin-3 is frequently associated with mortality, its independent prognostic value may decrease after adjustment for established markers such as NT-proBNP, soluble suppression of tumorigenicity 2 (sST2), or high-sensitivity cardiac troponin T (hs-cTnT). Therefore, Galectin-3 appears most useful as a complementary biomarker within a multimarker strategy rather than as an isolated prognostic tool.

In summary, Galectin-3 is relevant because it captures the inflammatory-fibrotic remodeling axis of heart failure, complementing NT-proBNP, which mainly reflects wall stress and hemodynamic overload. Its greatest clinical value lies in integration into multimarker models aimed at improving disease phenotyping and prognostic stratification.

### 1.6. Conceptual Framework—A Redox–Galectin-3–Fibrosis–Imaging Model of Heart Failure

Heart failure may be viewed as a redox–inflammatory–fibrotic continuum in which mitochondrial dysfunction, NADPH oxidases, and xanthine oxidase promote oxidative injury, disturbed calcium handling, endothelial dysfunction, inflammation, and fibroblast activation. Persistent redox imbalance may therefore contribute to the transition from cellular stress to maladaptive extracellular matrix remodeling [5,45,46].

Within this system, Galectin-3 represents both a context-dependent fibro-inflammatory mediator and a circulating biomarker of remodeling. Experimental data support its involvement in macrophage activation, fibroblast proliferation, collagen deposition, and profibrotic signaling, whereas clinical studies associate elevated Galectin-3 with heart failure with preserved ejection fraction (HFpEF), diastolic dysfunction, right ventricular impairment, and adverse outcomes. However, circulating Galectin-3 should not be interpreted as a direct measure of myocardial fibrosis because it is also influenced by renal function, systemic inflammation, and comorbidities [36,38,39,47].

Biochemical biomarkers and imaging provide complementary information. Redox markers, such as MDA, 8-OHdG, MPO, and circulating thiols, reflect oxidative injury or antioxidant reserve; NT-proBNP reflects hemodynamic stress; and Galectin-3 reflects fibro-inflammatory remodeling. Echocardiography identifies the functional consequences of remodeling through parameters such as the ratio of early mitral inflow velocity to early diastolic mitral annular velocity (E/e′), global longitudinal strain (GLS), left atrial strain, tricuspid annular plane systolic excursion (TAPSE), and the TAPSE/pulmonary artery systolic pressure (PASP) ratio, whereas cardiac magnetic resonance provides tissue-level characterization by late gadolinium enhancement, native T1 mapping, and extracellular volume fraction. Their integration may improve heart failure phenotyping and risk stratification, although prospective multimodal validation remains necessary [3,25,32,48,49,50,51].

**Figure 1:**
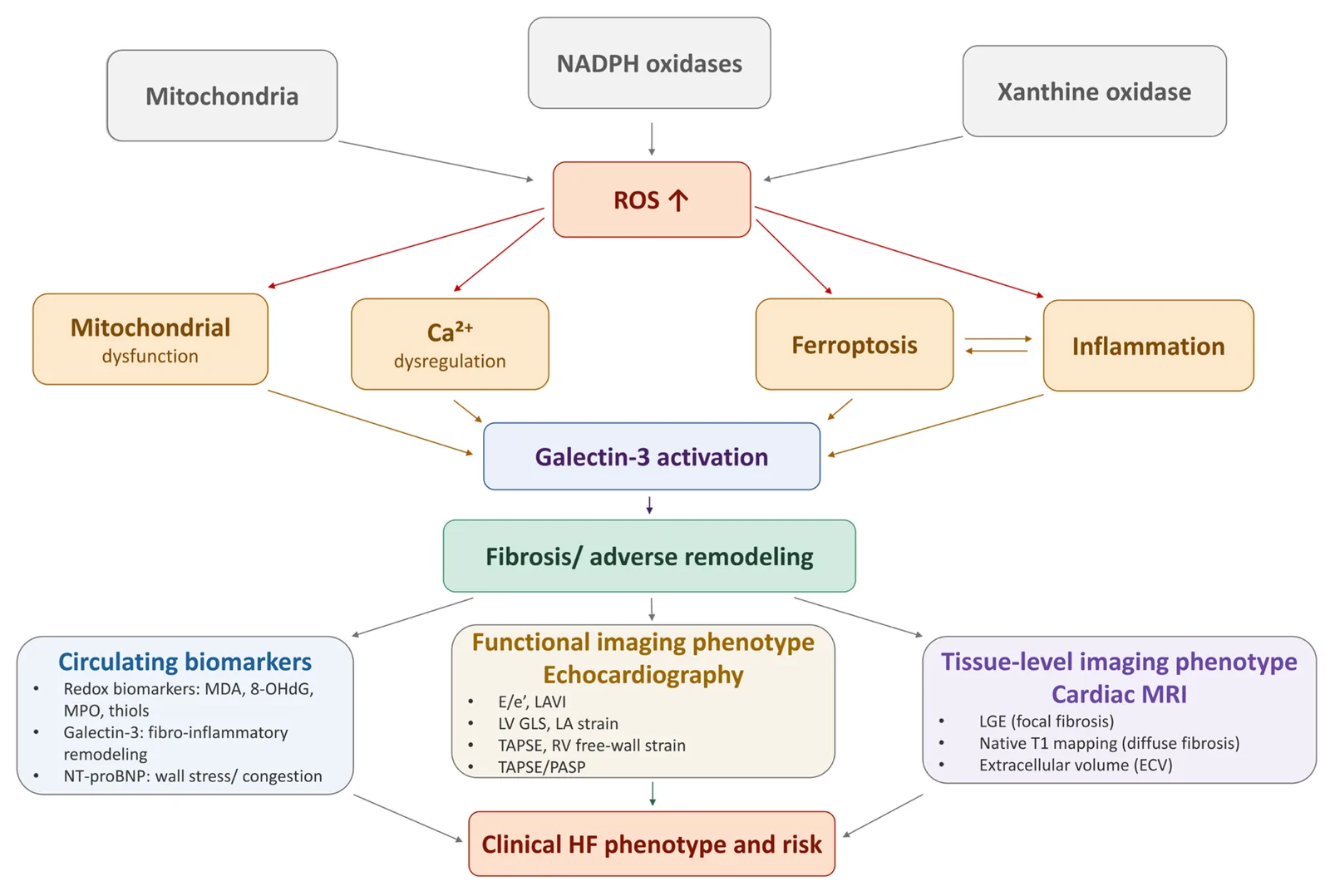
Integrated redox–Galectin-3–fibrosis–imaging model in heart failure. ROS, reactive oxygen species; NADPH, nicotinamide adenine dinucleotide phosphate; MDA, malondialdehyde; 8-OHdG, 8-hydroxy-2′-deoxyguanosine; MPO, myeloperoxidase; NT-proBNP, N-terminal pro-B-type natriuretic peptide; E/e′, ratio of early mitral inflow velocity to early diastolic mitral annular velocity; LAVI, left atrial volume index; LV, left ventricular; GLS, global longitudinal strain; LA, left atrial; TAPSE, tricuspid annular plane systolic excursion; RV, right ventricular; PASP, pulmonary artery systolic pressure; LGE, late gadolinium enhancement; ECV, extracellular volume fraction; MRI, magnetic resonance imaging; HF, heart failure.

This integrated framework is illustrated in Figure 1.

The novelty of this review, therefore, is not in considering oxidative stress, Galectin-3, and imaging abnormalities as separate topics, but in proposing their integration into a unified redox–fibrosis–imaging continuum. This approach may help refine heart failure phenotyping and identify biologically distinct subgroups for future multimarker and precision-oriented studies.

### 1.7. Literature Search Strategy

This narrative review was based on a structured literature search conducted in the PubMed/MEDLINE and Scopus databases, primarily covering publications from January 2015 to March 2026. Selected earlier landmark studies were included when directly relevant to the mechanistic or clinical framework. The search included the following terms, used alone or in combination: “heart failure”, “oxidative stress”, “reactive oxygen species”, “galectin-3”, “cardiac remodeling”, “fibrosis”, “ferroptosis”, “NT-proBNP”, “redox biomarkers”, “HFpEF”, “sodium–glucose cotransporter 2 (SGLT2) inhibitors”, “right ventricular dysfunction”, “malondialdehyde”, “thiol”, “8-hydroxy-2′-deoxyguanosine”, and “diastolic dysfunction”. Additional relevant studies were identified through manual screening of the reference lists of selected articles. Only articles published in English were considered. Given the narrative nature of this review, studies were selected based on their relevance to the topic, their methodological quality, and their contribution to the mechanistic and clinical understanding of oxidative stress in heart failure.

## 2. Molecular Mechanisms: The “Engine” of Disease

### 2.1. Mitochondrial Dysfunction—The Central Node of Oxidative Stress

Mitochondrial dysfunction represents one of the central links in the progression of heart failure, exceeding the significance of a simple secondary energy deficit and constituting an active pathogenic process. As emphasized by Liu et al. [45], the mitochondrion is one of the main intracellular sources of reactive oxygen species, generated particularly through alteration of the respiratory chain. The accumulation of mitochondria-derived ROS disrupts calcium (Ca^2+^) homeostasis, impairs energy metabolism, and activates cell death pathways, thereby directly contributing to the progressive deterioration of myocardial function.

This perspective has led to the emergence of the idea that the mitochondrion should be interpreted as a major determinant of disease progression. Several therapeutic strategies aim to preserve mitochondrial function or improve mitochondrial bioenergetics. In a comprehensive review, Maejima [52] summarizes preclinical and clinical data suggesting that SGLT2 inhibitors may exert cardioprotective effects also through mechanisms related to mitochondrial function. The author describes several possible pathways involved, including increased ketone body utilization, reduction in intracellular sodium and calcium overload, improvement of antioxidant defense, and possible modulation of mitochondrial dynamics through fusion and fission processes. Although many of these mechanisms remain incompletely elucidated, the review supports the idea that the benefits of SGLT2 inhibitors in heart failure extend beyond the simple reduction in hemodynamic load and may include an important component of mitochondrial protection.

The importance of altered mitochondrial dynamics is also illustrated by experimental data directly linking oxidative stress to mechanical dysfunction of the cardiomyocyte. Thus, Lozhkin et al. [53] demonstrated, in an experimental model of diastolic dysfunction, that NADPH oxidase 4 (NOX4)-mediated mitochondrial oxidative stress promotes mitochondrial fragmentation and disrupts the fusion–fission balance. The consequence of this subcellular remodeling is impairment of the mitochondria’s capacity to buffer Ca^2+^, with delayed clearance of calcium from the cytosol and altered cardiomyocyte relaxation. This mechanism is particularly relevant because it shows that diastolic dysfunction may arise not only through cell loss or advanced fibrosis, but also through early disturbances in the communication between oxidative stress, calcium homeostasis, and mitochondrial bioenergetics.

A key link in the progression of heart failure is mitochondrial Ca^2+^ overload, which links calcium homeostasis dysregulation to oxidative stress and bioenergetic impairment. Johnson et al. [54] reviewed evidence showing that abnormal Ca^2+^ release from the sarcoplasmic reticulum through ryanodine receptor 2 (RyR2) receptors increases cytosolic calcium concentration and forces mitochondria to take up excessive amounts of Ca^2+^. This mitochondrial overload promotes increased production of reactive oxygen species and compromises mitochondrial function, suggesting that cardiac dysfunction, including diastolic dysfunction, reflects the consequence of a subcellular disturbance of the Ca^2+^–redox–energetic axis.

Experimental data from Santulli et al. [55] showed that diastolic Ca^2+^ leak through RyR2 induces mitochondrial Ca^2+^ overload, mitochondrial dysfunction, and increased ROS production in post-infarction heart failure.

Their findings support a self-amplifying mechanism: mitochondrial oxidative stress promotes post-translational modifications of RyR2, including its oxidation, which further amplifies Ca^2+^ loss from the sarcoplasmic reticulum. Through this mechanism, energetic dysfunction is perpetuated, and post-ischemic cardiac dysfunction is aggravated.

The bidirectional relationship between mitochondrial Ca^2+^ homeostasis, redox status, and energy metabolism is further explored by Cortassa et al. [56]. The authors discuss data derived from models with “leaky” RyR, in which mitochondrial Ca^2+^ overload is associated with increased oxidative stress, mitochondrial membrane depolarization, and reduced adenosine triphosphate (ATP) content or synthesis. In addition, through computational modeling, they suggest that the impact of mitochondrial Ca^2+^ on ROS production and energetics depends on the redox environment and intracellular sodium (Na^+^). This interpretation supports the idea that cardiac dysfunction is deeply linked to the pathological interaction between dysregulated calcium homeostasis, redox imbalance, and impaired energy metabolism.

The importance of these mechanisms becomes even more evident in phenotypes dominated by myocardial stiffness and diastolic dysfunction. In this regard, Yue et al. [57] emphasize, in the context of HFpEF, that mitochondrial dysfunction represents one of the major causes of increased myocardial stiffness, through amplification of ROS production and upregulation of NOX4. Yue et al. [57] linked increased lipid peroxidation markers, such as malondialdehyde (MDA), with the development of oxidative stress, fibroblast activation, and excessive collagen deposition, processes that promote progressive interstitial remodeling and the development of the diastolic dysfunction characteristic of this phenotype.

This link between oxidative stress and fibrotic remodeling is reinforced by Teuber et al. [46], who highlight the important role of NOX4 in cardiac fibrotic remodeling. The authors show that this isoform is upregulated in cardiac fibroblasts in the setting of heart failure and in response to transforming growth factor beta (TGF-β), and that NOX4-generated ROS are necessary for activation of the small mothers against decapentaplegic 2/3 (Smad2/3) pathway, transformation of fibroblasts into myofibroblasts, and increased collagen production. Therefore, oxidative stress does not merely accompany myocardial remodeling, but actively participates in myocardial stiffening and progression of diastolic dysfunction.

Beyond primary mitochondria-derived reactive oxygen species (mtROS) generation, mitochondrial dysfunction may become self-amplifying through ROS-induced ROS release, loss of membrane potential, and mitochondrial DNA damage or release. Experimental evidence indicates that these events can connect redox imbalance with cyclic GMP–AMP synthase–stimulator of interferon genes (cGAS–STING), TANK-binding kinase 1 (TBK1), and interferon regulatory factor 3 (IRF3) signaling, inflammasome activation, and regulated cardiomyocyte death, with membrane permeabilization mediated by Bcl-2 homologous antagonist/killer (BAK) and Bcl-2-associated X protein (BAX), as well as voltage-dependent anion channel 1 (VDAC1)-dependent processes, proposed as mechanistic links. These pathways extend the redox–inflammatory continuum from mitochondrial injury to adverse remodeling; however, their relative contribution in human heart failure remains incompletely defined [58,59,60].

The fundamental concept integrating these mechanisms was previously systematized by Tsutsui et al. [5], who identified the main sources of ROS in heart failure—mitochondria, NAD(P)H oxidases, and xanthine oxidase—and showed that excess ROS activates kinases involved in hypertrophic signaling, such as mitogen-activated protein kinase (MAPK) and c-Jun N-terminal kinase (JNK), modifies proteins involved in excitation–contraction coupling, and stimulates fibroblast proliferation and matrix metalloproteinase activation. Through these effects, oxidative stress directly contributes to maladaptive myocardial remodeling.

Within this framework, Johnson et al. [54] complement the model proposed by Tsutsui by showing that mitochondrial oxidative stress contributes to the activation and transdifferentiation of cardiac fibroblasts. In cardiac fibroblasts, ROS-dependent activation of the p38 mitogen-activated protein kinase (p38-MAPK) pathway is associated with increased collagen synthesis and development of a profibrotic phenotype. Thus, the mitochondrion emerges as the meeting point between oxidative stress, disturbed calcium homeostasis, sterile inflammation, and structural remodeling.

Collectively, these data support the idea that mitochondrial dysfunction represents the central intermediary of the interaction between oxidative stress, Ca^2+^ dysregulation, and cardiac remodeling. It not only reflects myocardial injury, but actively participates in the self-amplification of pathological processes that lead to myocardial stiffening, fibrosis, inflammation, and, ultimately, to the progression of heart failure.

### 2.2. Ferroptosis—A Distinct Form of Regulated Cell Death Dependent on Oxidative Stress and Iron

If mitochondrial dysfunction represents one of the main sources of oxidative stress in heart failure, then ferroptosis can be regarded as one of the most relevant consequences of this redox imbalance. Unlike classical apoptosis, ferroptosis is a distinct form of regulated cell death characterized by iron dependence and by the lethal accumulation of lipid peroxidation at the level of cellular membranes [61].

This interpretation was experimentally supported by Fang et al. [62], who demonstrated that, in murine models of doxorubicin-induced cardiomyopathy and ischemia/reperfusion injury, activation of the nuclear factor erythroid 2-related factor 2 (Nrf2)/heme oxygenase 1 (Hmox1) axis promotes heme degradation and release of free iron. This excess iron accumulates particularly within mitochondria, where it promotes the initiation of lipid peroxidation and cardiomyocyte death through ferroptosis. Importantly, blockade of this mechanism with ferrostatin-1 or iron chelation with dexrazoxane significantly reduced myocardial injury and attenuated cardiac remodeling, suggesting that ferroptosis acts as an important effector mechanism of disease progression.

At the mechanistic level, ferroptosis is initiated when iron dyshomeostasis and ROS generation exceed the capacity of antioxidant systems, particularly the cystine/glutamate antiporter system xc− (System xc−)/glutathione (GSH)/glutathione peroxidase 4 (GPX4) axis, leading to uncontrolled peroxidation of polyunsaturated fatty acid-containing membrane phospholipids. Mitochondria may amplify this process through iron accumulation, altered redox metabolism, and lipid peroxide formation, whereas impaired GPX4 activity prevents detoxification of lipid hydroperoxides. Across experimental models of ischemic, diabetic, and anthracycline-related cardiomyopathy, these mechanisms have been associated with cardiomyocyte loss and adverse ventricular remodeling. Although iron chelators, ferroptosis inhibitors, and restoration of antioxidant pathways show cardioprotective effects in preclinical models, their role in human heart failure is still unproven [62,63,64,65,66]. Recent reviews also emphasize that ferroptosis may promote myocardial fibrosis through iron dysregulation, lipid peroxidation, and impairment of GSH/GPX4-dependent antioxidant defenses, thereby linking cardiomyocyte injury to fibroblast activation and extracellular matrix remodeling; however, most therapeutic evidence remains preclinical [67].

In HFpEF, persistent oxidative stress may increase susceptibility to ferroptosis through disruption of the Nrf2/system xc−/GSH–GPX4 antioxidant network. This disruption provides a plausible mechanistic link between redox imbalance, lipid peroxidation, cardiomyocyte injury, and myocardial stiffness. However, evidence specific to human HFpEF remains limited and is currently derived predominantly from mechanistic and experimental studies [68].

Ferroptosis may also interact with inflammatory cell-death programs. In pressure-overload heart failure, acyl-CoA synthetase long-chain family member 4 (ACSL4)-associated ferroptosis has been linked to NOD-like receptor family pyrin domain containing 3 (NLRP3) inflammasome activation, caspase-1 activation, and interleukin-1 beta (IL-1β) maturation, while broader experimental evidence suggests that lipid peroxidation and GPX4 impairment can facilitate damage-associated molecular pattern (DAMP)-mediated innate immune signaling. This ferroptosis–pyroptosis crosstalk provides a mechanistic route through which redox injury may be converted into self-sustaining necroinflammation and adverse remodeling; its quantitative relevance in human heart failure is still unclear [69,70].

Collectively, mechanistic, experimental, and translational evidence indicates that mitochondrial dysfunction is a key component of oxidative stress in heart failure, linking Ca^2+^ dysregulation, impaired bioenergetics, proinflammatory signaling, and maladaptive structural remodeling [5,45,46,58,60]. Building on this framework, ferroptosis emerges as an important downstream consequence of redox imbalance, connecting iron overload, GSH/GPX4 depletion, lipid peroxidation, cardiomyocyte death, and inflammatory activation [62,63,64,68,69,70].

## 3. Circulating Biomarkers

### 3.1. Biomarkers of Oxidative Injury (MDA, 8-OHdG) and Antioxidant Capacity (Thiols)

If the molecular mechanisms discussed above place oxidative stress at the center of heart failure pathogenesis, the next logical step is to assess how these processes can be captured systemically through circulating biomarkers. A fundamental question is therefore whether redox imbalance can be reproducibly demonstrated in the circulation and differentiated from the healthy population. Available data suggest that the answer is yes, and that markers of oxidative injury, such as malondialdehyde (MDA) and 8-hydroxy-2′-deoxyguanosine (8-OHdG), together with markers of antioxidant reserve such as thiol groups, may reflect not only the presence of heart failure, but also its severity, etiologic substrate, and the degree of associated ischemic burden [26,71].

#### 3.1.1. Global Population-Level Evidence

At the population level, meta-analyses consistently support the presence of a systemic oxidative profile in heart failure. Milani et al., in a systematic review and meta-analysis including 3015 patients with heart failure and 2704 healthy controls, reported higher concentrations of oxidative injury markers, particularly 8-OHdG and MDA, together with shorter telomere length. Earlier pooled evidence from Di Minno et al. similarly showed increased 8-OHdG levels in patients with heart failure, measured in plasma or urine, with higher values generally associated with more advanced functional limitation. Taken together, these data support the concept that heart failure is accompanied by persistent systemic oxidative injury rather than by an isolated myocardial redox disturbance [26,28].

#### 3.1.2. Cross-Sectional Clinical Validation

Cross-sectional studies provide patient-level confirmation of the systemic oxidative profile observed in meta-analyses. Rivera et al. reported higher levels of 8-OHdG and lipid peroxidation products in patients with heart failure than in healthy controls, with the highest values observed in hypertensive cardiomyopathy. Similarly, Kobayashi et al. showed that urinary 8-OHdG is increased in chronic heart failure and rises with worsening NYHA functional class; its associations with left ventricular ejection fraction, pulmonary capillary wedge pressure, and indexed left ventricular end-diastolic volume further support a relationship between oxidative injury and disease severity. The higher concentration of 8-OHdG in coronary sinus blood than in the aortic root also suggests that the failing myocardium may contribute directly to systemic oxidative stress. However, these cross-sectional findings do not establish biomarker thresholds for routine clinical use or prove causal directionality [72,73].

#### 3.1.3. Etiologic Stratification: Ischemic vs. Non-Ischemic

Etiology modifies the clinical interpretation of redox biomarkers in heart failure. In cohorts comparing ischemic with non-ischemic cardiomyopathy, absolute MDA and uric acid levels showed greater prognostic relevance in ischemic disease, whereas the MDA/protein sulfhydryl groups (PSH) ratio appeared as a stronger predictor in non-ischemic cardiomyopathy. Differences in MDA, PSH, and the MDA/PSH ratio, together with their associations with NYHA class, left ventricular ejection fraction (LVEF), maximal oxygen uptake (VO_2_max), and NT-proBNP, indicate that oxidative injury and antioxidant reserve should be interpreted in the context of heart failure etiology. Composite indices integrating oxidative damage with antioxidant reserve may more precisely capture net redox status than isolated biomarkers; however, external validation is required before etiology-specific thresholds can be proposed [27,74].

This heterogeneity may partly reflect the ischemic substrate itself, as higher MDA levels have also been associated with greater angiographic coronary artery disease burden in non-heart-failure cohorts [75,76]. In patients with chronic coronary syndrome, routine erythrocyte and platelet distribution indices have also been explored as correlates of angiographic coronary lesion extent; however, their relevance to heart-failure redox phenotyping remains indirect and requires dedicated validation [77].

#### 3.1.4. Thiols—Biomarkers of Antioxidant Reserve Depletion

Serum thiols provide information complementary to markers of oxidative injury by reflecting depletion of systemic antioxidant reserve. In the multicenter BIOSTAT-CHF cohort, lower free thiol concentrations were associated with more advanced NYHA class, higher NT-proBNP values, and increased all-cause and cardiovascular mortality, supporting their potential value as a prognostic redox marker distinct from hemodynamic stress. Smaller studies in patients with advanced or exacerbated heart failure similarly reported reduced total thiols and biological antioxidant potential together with increased derivatives of reactive oxygen metabolites, suggesting progressive exhaustion of endogenous antioxidant defenses. Taken together, thiol-based markers may help capture the balance between oxidative injury and antioxidant capacity; however, assay standardization and clinically validated thresholds are necessary before routine implementation [32,33].

#### 3.1.5. Multimarker Integration: Redox + Hemodynamic

Multimarker assessment may improve risk stratification by combining information on hemodynamic stress with markers of systemic redox imbalance. In patients with heart failure with reduced ejection fraction (HFrEF), Gtif et al. developed a generalized linear model in which total antioxidant capacity and uric acid, together with NT-proBNP, improved discrimination of post-discharge mortality compared with NT-proBNP alone. Complementary evidence from Park et al. in acute heart failure showed that patients with concomitantly elevated uric acid and NT-proBNP had the highest 3-month risk of cardiac death or rehospitalization. These data support the concept that altered oxidative metabolism and hemodynamic burden represent complementary biological dimensions of heart failure. However, current models remain cohort- and assay-dependent and do not yet define a standardized redox–hemodynamic panel for routine clinical use [24,29].

#### 3.1.6. HFpEF-Specific Features

HFpEF is characterized by diastolic dysfunction, myocardial stiffness, and interstitial remodeling in a clinical context frequently dominated by metabolic comorbidity, systemic inflammation, and microvascular endothelial dysfunction. Within this phenotype, oxidative and nitrosative stress may promote reduced nitric oxide bioavailability, impaired cyclic guanosine monophosphate–protein kinase G (cGMP–PKG) signaling, cardiomyocyte stiffening, and extracellular matrix remodeling. However, currently available circulating redox markers should be interpreted as phenotype-associated indicators rather than disease-specific diagnostic tools [78].

Clinical data support the presence of impaired antioxidant reserve and increased nitrosative stress in HFpEF. Turinay Ertop et al. reported lower native and total thiol concentrations in patients with HFpEF, with inverse associations with NT-proBNP and carbohydrate antigen 125 (CA-125), suggesting that reduced antioxidant capacity accompanies the hemodynamic and congestive burden of the disease. Additional data from Momot et al. showed higher circulating 3-nitrotyrosine (3-NT) concentrations in HFpEF than in healthy controls and HFrEF, supporting a distinct nitrosative–oxidative profile [34,79]. In a perioperative cohort undergoing cardiopulmonary bypass, patients with preoperative HFrEF showed higher plasma and atrial levels of MDA and 3-NT, together with increased atrial NLRP3 expression, compared with those with HFpEF; however, these observations should be interpreted within the specific context of surgical and ischemia–reperfusion-related injury [80].

The available evidence suggests that HFpEF may be associated with a distinct redox phenotype characterized by impaired antioxidant reserve, increased nitrosative stress, endothelial dysfunction, and myocardial stiffening. These mechanisms may contribute to the transition from systemic cardiometabolic inflammation to diastolic dysfunction and interstitial remodeling. However, current redox biomarkers remain complementary and phenotype-associated rather than disease-specific diagnostic tools, and prospective studies are required to define their prognostic value and possible role in multimarker phenotyping [34,78,79].

Overall, the available clinical evidence indicates that oxidative stress in heart failure is a systemic, quantifiable, and clinically relevant pathogenic dimension rather than a simple biochemical epiphenomenon. Markers of oxidative injury, including MDA and 8-OHdG, support the presence of persistent redox damage, whereas thiol-based measures show depletion of endogenous antioxidant reserve. Their associations with clinical severity, NT-proBNP, outcome, etiology, and HFpEF-related phenotypes support a complementary role in redox phenotyping. However, inter-assay variability, differences in patient populations, and the absence of validated thresholds currently limit routine clinical implementation [26,27,32,34,74,79].

### 3.2. Galectin-3 at the Redox–Inflammation–Fibrosis Interface

If redox biomarkers such as MDA, 8-OHdG, and thiols mainly capture the intensity of biochemical injury and the capacity of the antioxidant system to counteract it, Galectin-3 (Gal-3) adds an additional dimension, namely the structural expression of these processes, represented by fibrotic remodeling. Thus, Gal-3 may be regarded as a mechanistically relevant biomarker situated at the interface between inflammation, oxidative stress, and fibrogenesis, reflecting the transition from molecular imbalance to maladaptive myocardial remodeling [81].

#### 3.2.1. Clinical and Prognostic Relevance: Association Beyond Hemodynamic Stress

Clinical studies consistently associate higher circulating Galectin-3 concentrations with adverse outcomes in heart failure. In a meta-analysis of 24 cohorts, Cheng et al. reported a dose–response relationship between Galectin-3 levels and long-term all-cause mortality. Similarly, in adults presenting with acute dyspnea, Zwawi et al. found that Galectin-3 at presentation was associated with 30-day all-cause mortality after adjustment for NT-proBNP and clinical predictors, although its incremental prognostic value was modest. This evidence suggests that Galectin-3 captures biological information not fully represented by markers of hemodynamic stress.

However, interpretation of circulating Galectin-3 requires caution. Its concentration may be influenced by renal dysfunction, systemic inflammation, and comorbidity, and it should not be regarded as a direct measure of myocardial fibrosis. Accordingly, Galectin-3 is best viewed as a complementary fibro-inflammatory biomarker within a multimarker strategy, rather than as a standalone prognostic tool or a substitute for NT-proBNP or imaging-based tissue characterization [36,44,82,83].

#### 3.2.2. Relevance in HFpEF: Association with Diastolic Dysfunction and Risk

HFpEF is characterized by diastolic dysfunction, myocardial stiffness, and interstitial remodeling in a phenotype frequently associated with systemic inflammation, oxidative stress, and metabolic comorbidity. In this setting, higher circulating Galectin-3 concentrations have been associated with HFpEF and with echocardiographic indices of impaired filling, including E/e′. Meta-analytic evidence also links higher Galectin-3 levels with adverse outcomes; however, heterogeneity among cohorts and the influence of renal dysfunction and comorbidity preclude interpreting circulating Galectin-3 as a direct measure of myocardial fibrosis or ventricular stiffness [36,38].

Prospective data from the Diastolic Dysfunction in Heart Failure (DIAST-CHF) study support a complementary rather than replacement role for Galectin-3. In individuals with cardiovascular risk factors, Galectin-3 was associated with incident HFpEF and long-term adverse outcomes, whereas NT-proBNP showed stronger predictive performance for incident HFpEF. Thus, Galectin-3 may enrich fibro-inflammatory phenotyping in patients with suspected or established HFpEF when assessed alongside natriuretic peptides and echocardiographic findings, but no Galectin-3-based diagnostic threshold or standalone clinical pathway has been validated [3,84].

#### 3.2.3. Mechanistic Basis: Galectin-3 at the Redox–Inflammation–Fibrosis Interface

Galectin-3 is positioned at the interface between macrophage activation, fibroblast signaling, and extracellular matrix remodeling. Experimental evidence indicates that extracellular Galectin-3 can regulate immune and stromal cell behavior, promote fibroblast-to-myofibroblast transition, and enhance collagen deposition. These actions provide a mechanistic basis through which inflammatory and oxidative injury may be translated into persistent fibrotic remodeling. However, the biological effects of Galectin-3 are context- and time-dependent, and inhibition may have different consequences during acute tissue repair and chronic remodeling [39].

A more specific example is given by the cardiotrophin-1 (CT-1)/Galectin-3 axis. In human cardiac fibroblasts, CT-1 induces Galectin-3 expression through extracellular signal-regulated kinase 1/2 (ERK1/2) and signal transducer and activator of transcription 3 (STAT3) signaling, whereas experimental data indicate that Galectin-3 contributes to the resulting proinflammatory and profibrotic response. In hypertensive heart failure, higher CT-1 and Galectin-3 concentrations have also been associated with myocardial fibrosis and adverse outcomes. These results support an active signaling role for Galectin-3 in selected experimental and translational settings, whereas direct causal inference from circulating Galectin-3 measurements in human heart failure remains limited [85].

#### 3.2.4. Extension to the Right Ventricle: Association with RV Dysfunction

Higher Galectin-3 concentrations have also been associated with right ventricular dysfunction, notably in the presence of increased pulmonary afterload. In patients with HFrEF, higher Galectin-3 concentrations have been associated with lower tricuspid annular plane systolic excursion (TAPSE) and tricuspid annular systolic velocity (s′), higher pulmonary artery systolic pressure (PASP), and impaired right ventricular–pulmonary arterial (RV–PA) coupling assessed by the TAPSE/PASP ratio. Additional cohort data suggest that Galectin-3 may provide complementary information on RV dysfunction when assessed alongside NT-proBNP. However, these associations do not establish Galectin-3 as a specific marker of right ventricular fibrosis or as a substitute for echocardiographic assessment. The redox-dependent mechanisms and imaging phenotype of right ventricular remodeling are discussed in more detail in Section 4.2 [47,86].

#### 3.2.5. Multimarker Integration: Complementary Prognostic Information

Galectin-3 may be most informative when interpreted within a multimarker strategy rather than as an isolated biomarker. In hospitalized heart failure, de Boer et al. showed that higher Galectin-3 concentrations were associated with an increased risk of death or heart failure hospitalization after adjustment for natriuretic peptides, renal function, and clinical covariates, with a more pronounced association in HFpEF. In stable chronic heart failure, Grande et al. further reported that combining NT-proBNP, soluble suppression of tumorigenicity 2 (sST2), and Galectin-3 improved 1-year prognostic discrimination compared with the use of each biomarker alone. The available evidence supports the concept that natriuretic peptides predominantly reflect hemodynamic stress, whereas Galectin-3 and sST2 may provide complementary information on fibro-inflammatory remodeling. However, differences in patient populations, assays, and risk thresholds mean that no universally validated Galectin-3-based multimarker panel is currently available for routine clinical use [87,88].

#### 3.2.6. Serial Galectin-3 Changes and Incident Heart Failure: Exploratory Evidence

Longitudinal population studies suggest that changes in Galectin-3 may reflect subclinical fibro-inflammatory processes preceding overt heart failure. In the Framingham Heart Study, an increase in Galectin-3 over approximately 10 years was associated with a higher risk of incident heart failure, cardiovascular disease, and all-cause mortality after adjustment for baseline values and conventional risk factors. Similarly, in the Atherosclerosis Risk in Communities (ARIC) cohort, elevated midlife Galectin-3 concentrations and subsequent increases over time were associated with incident heart failure and mortality, although these associations were attenuated after adjustment for NT-proBNP and high-sensitivity cardiac troponin (hs-cTn). The results support serial Galectin-3 assessment as a potential research tool for risk phenotyping, but they do not establish a validated monitoring strategy, therapeutic target, or clinical decision threshold [89,90].

#### 3.2.7. From Association to Causality: Experimental Evidence

Experimental models support a causal but context-dependent role of Galectin-3 in redox–fibrotic remodeling. In doxorubicin-related cardiac injury, increased myocardial Galectin-3 expression has been associated with oxidative stress, interstitial fibrosis, and ventricular dysfunction, whereas Galectin-3 suppression or deficiency reduced oxidative injury and remodeling in selected models. However, this effect is not uniformly detrimental. In an acute doxorubicin model, early Galectin-3 deficiency aggravated myocardial injury, oxidative stress, depletion of glutathione and catalase, and mortality, suggesting that Galectin-3 may have a role in acute stress responses and tissue repair [39,91].

Additional genetic and pharmacological studies support a pro-remodeling role for Galectin-3 during chronic injury. In a desmin-deficiency model of progressive heart failure, Galectin-3 deletion reduced inflammation, profibrotic gene expression, adverse remodeling, and long-term systolic and diastolic dysfunction. Similarly, pharmacological inhibition with the Galectin-3 C-terminal domain (Gal-3C) after myocardial infarction reduced fibrosis, scar expansion, ventricular dilation, and inflammatory profibrotic signaling in experimental models. These findings support Galectin-3 as a possible therapeutic target in chronic fibro-inflammatory remodeling; however, they remain preclinical and cannot establish therapeutic benefit in human heart failure [92,93].

Current evidence supports a dual but non-equivalent role of Galectin-3 in heart failure. Experimental studies indicate that Galectin-3 can actively participate in macrophage–fibroblast crosstalk, extracellular matrix deposition, and oxidative–inflammatory amplification, although these effects remain context- and time-dependent. In contrast, circulating Galectin-3 in clinical cohorts should primarily be interpreted as a complementary marker of fibro-inflammatory remodeling rather than as a direct measure of myocardial fibrosis or a validated causal surrogate. Its concentration may also be influenced by renal dysfunction, systemic inflammation, and comorbidity. Accordingly, Galectin-3 is most informative when used together with hemodynamic biomarkers, functional echocardiographic parameters, and CMR-based tissue characterization rather than as an isolated marker [3,36,39,50].

## 4. Clinical and Imaging Expression of Redox-Dependent Remodeling

### 4.1. Diastolic Dysfunction and HFpEF: Clinical and Imaging Correlates of Redox-Dependent Remodeling

One of the most important clinical expressions of redox-dependent remodeling is the development of diastolic dysfunction, particularly in the phenotype of heart failure with preserved ejection fraction (HFpEF). Available data suggest that oxidant–antioxidant imbalance does not appear only in advanced stages of disease, but may be identified early, already in phases in which ejection fraction remains preserved, while the dominant changes are represented by myocardial stiffening, concentric hypertrophy, and impaired ventricular relaxation.

Clinical evidence supports impaired antioxidant reserve as a relevant component of the HFpEF phenotype. In hypertensive HFpEF, Turinay Ertop et al. reported lower native and total thiol concentrations, with inverse associations with NT-proBNP and CA-125, suggesting that reduced antioxidant capacity accompanies hemodynamic stress and congestion. Complementary evidence from the BIOSTAT-CHF cohort showed that lower free thiol concentrations were associated with more advanced clinical severity, higher NT-proBNP values, and adverse outcomes. Taken together, these data support thiol-based markers as complementary indicators of redox imbalance and disease burden, although they do not prove causality or validated thresholds for routine clinical use [32,34].

Beyond overt clinical forms, the relationship between oxidative stress and impaired myocardial compliance may also be captured in earlier, apparently preclinical stages. In this regard, Raad et al. [94], in the Women’s Ischemia Syndrome Evaluation–Coronary Vascular Dysfunction (WISE-CVD) cohort, investigated women with ischemia and no obstructive coronary artery disease (INOCA), that is, a group located in an intermediate zone between apparently preserved function and early diastolic impairment. Although these women had preserved ejection fraction and epicardial arteries without significant stenoses, 61% had elevated left ventricular end-diastolic pressure (LVEDP), suggesting the presence of subclinical diastolic dysfunction. Elevated plasma cystine levels, the oxidized form of cysteine, were independently associated with increased LVEDP and reduced peak filling rate measured by cardiac magnetic resonance. The lack of a similar correlation for glutathione suggests that extracellular markers of oxidative stress may more faithfully reflect the redox imbalance involved in early diastolic stiffening. Thus, systemic oxidative stress appears associated with impaired myocardial relaxation even before overt heart failure develops.

In the same direction, the study published by Aldiwani et al. [95], also within WISE-CVD, provides important support for the hypothesis of a link between coronary microvascular dysfunction and the development of HFpEF. The authors compared women with INOCA, patients with HFpEF, and control subjects using stress CMR, and observed a gradual reduction in myocardial perfusion reserve index (MPRI), with the lowest values observed in the HFpEF group, followed by the INOCA group. These data suggest that reduced myocardial perfusion represents a shared pathophysiological feature of INOCA and HFpEF. However, after adjustment for age, the differences were attenuated, with only the epicardial component of MPRI remaining significant. Therefore, the study does not demonstrate a direct causal continuum between the two entities, but supports the hypothesis that chronic microvascular ischemia may contribute to ventricular remodeling and to the emergence of the HFpEF phenotype in interaction with systemic cardiovascular risk factors.

The importance of oxidative stress in the early stages of diastolic remodeling becomes even more evident in populations with increased metabolic risk. In this regard, Oksen and Aslan [96] investigated patients with type 2 diabetes mellitus without known cardiovascular disease and reported significantly higher total oxidant status (TOS) and oxidative stress index (OSI) values and lower total antioxidant status (TAS) values in patients with type 2 diabetes than in controls. From an imaging point of view, the myocardial performance index derived by tissue Doppler was significantly higher in diabetic patients, in the absence of any reduction in ejection fraction. Moreover, the significant positive correlation between OSI and mitral MPI, present only in the diabetic group, suggests that oxidant–antioxidant imbalance is directly linked to impaired global myocardial performance already in the subclinical phase, before overt heart failure develops.

These observations are biologically plausible in diabetes, where chronic hyperglycemia, lipotoxicity, and altered fatty acid oxidation promote mitochondrial ROS generation and activate pathways involving advanced glycation end products (AGEs) and the receptor for advanced glycation end products (RAGE), protein kinase C (PKC), and the polyol and hexosamine pathways. These processes may contribute to myocardial hypertrophy, fibrosis, increased stiffness, and subclinical diastolic dysfunction before overt HF develops [97].

At the mechanistic level, the link between oxidative stress and diastolic stiffness is reinforced by the experimental study performed by Lozhkin et al. [53]. Using a murine model defined by increased mitochondrial oxidative stress, the authors demonstrated that excess mitochondrial ROS causes diastolic dysfunction in the absence of any major reduction in ejection fraction. The proposed mechanism involves altered mitochondrial dynamics and disruption of Ca^2+^ homeostasis, with consequent impairment of active myocardial relaxation. These data suggest that the diastolic stiffness observed echocardiographically in HFpEF may represent the clinical expression of mitochondrial energetic disturbance induced by oxidative stress.

An integrative perspective on this model is provided by Kumar et al. [98], who review the evidence on the role of mitochondrial dysfunction in HFpEF. The authors emphasize that exercise intolerance, the defining clinical sign of this phenotype, results from both cardiac and peripheral abnormalities, and that mitochondrial impairment may significantly contribute to defective oxygen utilization [99]. At the peripheral level, the cited studies highlight reduced mitochondrial content, impaired oxidative capacity, and altered muscle energetics in skeletal muscle, while at the myocardial level reduced energetic reserves and bioenergetic changes compatible with impaired mitochondrial function have been described. In addition, the review discusses the hypothesis that increased ROS and disturbed mitochondrial bioenergetics contribute to remodeling, inflammation, and diastolic dysfunction in HFpEF. Therefore, mitochondrial function appears not only as a mechanistic determinant of disease, but also as a promising therapeutic target, although the impact of mitochondrial interventions on symptoms and prognosis still requires additional validation.

In summary, clinical, imaging, and experimental data converge toward the same conclusion: the clinical expression of redox-dependent remodeling in HFpEF is dominated by diastolic dysfunction, myocardial stiffness, and exercise intolerance, and these phenomena may be detected early, before deterioration of ejection fraction. Redox biomarkers, markers of microvascular perfusion, and imaging parameters of diastolic function should not be viewed separately, but as complementary expressions of the same pathogenic process, in which oxidant–antioxidant imbalance, mitochondrial dysfunction, and fibrotic remodeling mutually reinforce one another and progressively lead to the full clinical HFpEF phenotype.

### 4.2. Ventricular Remodeling Across Heart Failure Phenotypes and Human Models of Oxidative Injury

Oxidative stress-dependent remodeling can occur in both ventricles and in different clinical settings. In chronic heart failure, it is expressed by left ventricular diastolic dysfunction, right ventricular–pulmonary arterial uncoupling, and progressive fibro-inflammatory remodeling. Anthracycline-associated cardiotoxicity provides a complementary human model in which oxidative myocardial injury and the temporal behavior of redox- and fibrosis-related biomarkers can be assessed before the onset of overt heart failure.

#### 4.2.1. Right Ventricular Remodeling and RV–PA Coupling

Right ventricular dysfunction in heart failure may not be explained solely by increased pulmonary afterload. In HFrEF, higher circulating Galectin-3 concentrations have been associated with lower TAPSE and tricuspid annular s′ velocity, higher pulmonary artery systolic pressure, more severe tricuspid regurgitation, and impaired RV–PA coupling assessed by TAPSE/PASP. Additional cohort data suggest that Galectin-3 may provide complementary information on right ventricular dysfunction when interpreted alongside NT-proBNP. These associations are consistent with a fibro-inflammatory component of right-heart remodeling, but they do not establish Galectin-3 as a specific marker of right ventricular fibrosis or as a substitute for echocardiographic assessment [47,86].

From a mechanistic perspective, the right ventricle may be particularly vulnerable to redox-dependent remodeling when chronic pressure overload is coupled with mitochondrial energetic stress. Thus, echocardiographic abnormalities such as reduced TAPSE, increased PASP, and impaired TAPSE/PASP should be interpreted as functional readouts of the right ventricle–pulmonary circulation unit, while the following subsection focuses on the mitochondrial mechanisms that may contribute to RV decompensation.

#### 4.2.2. Why the Right Ventricle Is Vulnerable: The Bioenergetic Substrate

Experimental evidence indicates that right ventricular failure under chronic pressure overload is not explained solely by increased afterload, but also by intrinsic mitochondrial and redox vulnerability. In murine pulmonary artery banding models, the transition from compensatory hypertrophy to decompensated right ventricular failure was associated with increased mitochondrial ROS generation, impaired oxidative phosphorylation, reduced bioenergetic efficiency, mitochondrial structural abnormalities, and activation of cell-death pathways. Hwang et al. further showed reduced expression and activity of electron transport chain complexes, impaired antioxidant defense including lower aldehyde dehydrogenase 2 activity, increased 4-hydroxynonenal, and disturbances in mitochondrial fission–fusion proteins such as mitochondrial fission factor (MFF) and optic atrophy 1 (OPA1). Together, these data support the concept that pressure-overload-induced RV failure involves a strong metabolic and redox substrate rather than a purely mechanical response [100,101].

Metabolic reprogramming may begin before overt RV decompensation. Experimental models of maladaptive RV hypertrophy show reduced glucose oxidation, increased glycolytic dependence, progressive RV ischemia, and suppression of oxidative metabolism, with activation of hypoxia-inducible factor 1-alpha (HIF-1α)- and Myc-related pathways. Thus, the transition from adaptive hypertrophy to RV failure may be viewed as a progressive mitochondrial bioenergetic crisis in which pressure overload, ischemia, ROS generation, and impaired energy production reinforce one another. Clinically, reduced TAPSE, increased PASP, and impaired TAPSE/PASP can be interpreted as functional manifestations of this adverse RV–pulmonary circulation phenotype [102].

#### 4.2.3. Left Ventricular Expression in HFpEF: Galectin-3 and Diastolic Dysfunction

The left ventricular expression of redox-dependent fibro-inflammatory remodeling is particularly relevant in HFpEF, where impaired myocardial compliance, diastolic dysfunction, and interstitial remodeling may precede overt systolic impairment. In a meta-analysis, Shi et al. reported that higher circulating Galectin-3 concentrations were associated with HFpEF and with echocardiographic indices of diastolic dysfunction, including E/e′ and deceleration time, as well as with adverse outcomes. In contrast, associations with geometric markers such as left ventricular mass index and left atrial volume index were less consistent. These observations suggest that circulating Galectin-3 may be more closely related to the functional consequences of remodeling than to isolated geometric measures [38].

A systematic review by Baccouche and Rhodenhiser similarly reported higher Galectin-3 concentrations in HFpEF than in control populations and described associations with diastolic dysfunction, ventricular stiffness, and clinical outcomes. However, heterogeneity in patient selection, renal function, comorbidity burden, and biomarker assays limits causal interpretation. Thus, in HFpEF, Galectin-3 should be viewed as a complementary fibro-inflammatory biomarker that may enrich interpretation of diastolic imaging phenotypes, rather than as a direct measure of myocardial fibrosis or ventricular stiffness [36,103].

#### 4.2.4. Anthracycline Cardiotoxicity as a Human Model of Time-Defined Oxidative Myocardial Injury

Anthracycline-associated cardiotoxicity provides a clinically relevant model in which oxidative myocardial injury is induced within a relatively defined temporal framework. In the Carvedilol Effect in Preventing Chemotherapy-Induced Cardiotoxicity (CECCY) trial, Wanderley Jr. et al. reported increases in myeloperoxidase (MPO) and Galectin-3 during chemotherapy, whereas higher baseline MPO concentrations were associated with a greater rise in troponin. Carvedilol attenuated troponin elevation only in women with MPO levels above the median, suggesting that pre-existing oxidative activation may modify individual susceptibility to subclinical myocardial injury. However, neither MPO nor Galectin-3 alone consistently predicted subsequent left ventricular ejection fraction decline [104].

Broader evidence supports MPO as a marker of early oxidative-inflammatory injury in anthracycline cardiotoxicity. Reviews and meta-analytic data indicate that MPO may increase after chemotherapy and that early changes in MPO, particularly when assessed with troponin, may help identify patients at higher risk of cardiotoxicity. In contrast, Galectin-3 has shown less consistent short-term predictive value, supporting the interpretation that it may be more relevant to later fibro-inflammatory remodeling than to acute oxidative injury. Although these observations cannot be directly extrapolated to chronic heart failure, they reinforce the principle that redox and fibrotic biomarkers may have separate temporal roles during myocardial injury [105,106].

#### 4.2.5. Conceptual Integration

Collectively, the available evidence indicates that redox-dependent remodeling in heart failure is not restricted to a single ventricular chamber. In the left ventricle, oxidative and inflammatory stress, together with Galectin-3-associated fibro-inflammatory signaling, may be expressed through impaired relaxation, increased myocardial stiffness, and altered deformation. In the right ventricle, chronic pressure overload may interact with mitochondrial ROS generation and metabolic failure, contributing to RV–PA uncoupling and progressive dysfunction. Although the timing and relative contribution of these mechanisms may differ across heart failure phenotypes, both ventricles may participate in a shared redox–inflammatory–fibrotic continuum [39,100,101].

Anthracycline-associated cardiotoxicity provides a complementary temporal model of oxidative myocardial injury. In this setting, MPO appears more closely related to early oxidative-inflammatory activation, whereas Galectin-3 may be more relevant to later fibro-inflammatory remodeling. Although these conclusions cannot be directly extrapolated to chronic heart failure, they support the concept that redox and fibrotic biomarkers may have distinct roles during myocardial injury and remodeling.

This framework does not imply that circulating Galectin-3 or redox biomarkers directly quantify biventricular fibrosis. Rather, they provide complementary biological context for imaging phenotypes. Supporting this integrative concept, in patients with a first acute myocardial infarction, higher circulating Galectin-3 concentrations were associated with impaired left ventricular global longitudinal strain and left atrial reservoir strain, while the combined Galectin-3–GLS model increased discrimination of in-hospital new-onset heart failure. However, the single-center design and short in-hospital follow-up require external validation [107]. Echocardiography identifies the functional expression of remodeling through indices of diastolic function, myocardial deformation, and RV–PA coupling, whereas CMR can further characterize focal scar and diffuse interstitial remodeling. Thus, integrated biomarker and imaging assessment may support a more complete phenotyping of heart failure than either approach alone [47,48,108].

### 4.3. Cardiac Magnetic Resonance as a Tissue-Level Imaging Phenotype of Fibrotic Remodeling

Cardiac magnetic resonance (CMR) provides a tissue-level perspective that supports circulating biomarkers and echocardiographic assessment in heart failure. While circulating redox biomarkers may indicate systemic oxidative injury or depletion of antioxidant reserve, and echocardiography captures the functional consequences of myocardial stiffness and altered ventricular mechanics, CMR can characterize focal scar and diffuse interstitial remodeling more directly. This distinction is particularly relevant in heart failure phenotypes in which structural remodeling may precede overt systolic dysfunction or may not be entirely captured by conventional echocardiographic parameters [3,48,49,108].

Late gadolinium enhancement (LGE) is primarily used to identify focal replacement fibrosis or scar. In ischemic heart disease, LGE commonly reflects infarct-related fibrosis, whereas in non-ischemic cardiomyopathies it may identify characteristic patterns of myocardial injury and replacement fibrosis. The presence and extent of LGE are generally associated with adverse remodeling, ventricular dysfunction, arrhythmic risk, and poorer clinical outcomes. However, LGE is less sensitive for diffuse interstitial fibrosis because it relies on contrast differences between abnormal and apparently normal myocardium [48,109,110].

Native T1 mapping and extracellular volume fraction (ECV) extend CMR tissue characterization beyond focal scar detection. Increased native T1 and ECV values may reflect diffuse interstitial expansion and extracellular matrix remodeling, particularly in non-ischemic cardiomyopathies and HFpEF-related myocardial stiffness. Nevertheless, these parameters should not be interpreted as direct fibrosis measurements in every clinical context, as native T1 and ECV may also be influenced by edema, infiltration, hematocrit, technical acquisition, and scanner-specific reference values. Their interpretation should therefore remain integrated with the clinical phenotype and other imaging findings [48,49,108,111].

The relationship between Galectin-3 and CMR-derived fibrosis markers remains clinically relevant but incompletely defined. In patients with non-ischemic dilated cardiomyopathy, higher circulating Galectin-3 levels have been associated with the presence of LGE, supporting a link between fibro-inflammatory activation and replacement fibrosis. However, the available evidence is limited by small cohorts, mixed populations, and the influence of renal dysfunction and systemic inflammation on Galectin-3 concentrations. Therefore, Galectin-3 provides complementary biological information and is not a surrogate for CMR tissue characterization [36,50].

From a translational perspective, CMR may provide the structural bridge between redox imbalance and the clinical expression of heart failure. Redox biomarkers may identify oxidative injury and impaired antioxidant reserve; Galectin-3 may indicate activation of fibro-inflammatory remodeling; and CMR may characterize the resulting myocardial substrate through LGE, native T1 mapping, and ECV. Echocardiography then provides the functional expression of this substrate through indices of diastolic dysfunction, myocardial deformation, atrial remodeling, and ventricular–vascular coupling. The merging of these domains may improve phenotyping and prognostic stratification, although prospective studies simultaneously assessing redox biomarkers, Galectin-3, CMR parameters, echocardiography, and clinical outcomes remain limited [3,4,108].

## 5. Clinical Applicability

From a clinical perspective, evaluation of heart failure should not be limited to a single biomarker. NT-proBNP remains the central marker of wall stress and hemodynamic overload, with established value for diagnosis, severity assessment, and prognosis. However, natriuretic peptides do not capture the full biological complexity of heart failure, including cardiomyocyte injury, inflammation, oxidative stress, fibro-inflammatory remodeling, and extracardiac organ dysfunction. Therefore, the most realistic clinical application of emerging biomarkers is not replacement of NT-proBNP, but its incorporation within a multimarker strategy that provides a more complete biological characterization of the disease [1,2,3,112,113]. Routine hematological indices may also provide complementary prognostic context: in a prospective cohort of patients hospitalized with decompensated heart failure, lower hemoglobin-to-red cell distribution width ratio was associated with worse unadjusted event-free survival, but did not retain independent prognostic value after adjustment for established clinical and biomarker covariates [114].

The potential value of this approach is supported by prognostic models integrating natriuretic peptides with markers of myocardial injury and clinical variables. In HFrEF, the integration of NT-proBNP and high-sensitivity cardiac troponin with a limited number of clinical variables has enabled robust and clinically applicable risk estimation. Conceptually, such panels are valuable because they capture complementary pathogenic domains: NT-proBNP reflects hemodynamic stress, troponin reflects cardiomyocyte injury, Galectin-3 reflects fibro-inflammatory remodeling, and redox biomarkers reflect oxidative injury or depletion of antioxidant reserve. However, the incremental value of individual biomarkers should be assessed in comparison to established clinical and biomarker-based risk models rather than considered in isolation [112,113,115].

Among non-traditional biomarkers, Galectin-3 may be particularly relevant in HFpEF and in patients with diastolic or right ventricular dysfunction. Higher circulating Galectin-3 concentrations have been associated with HFpEF, impaired diastolic indices, including E/e′, adverse outcomes, and markers of fibro-inflammatory remodeling. Nevertheless, Galectin-3 should not be interpreted as a direct measure of myocardial fibrosis or ventricular stiffness, because its circulating concentration is influenced by renal dysfunction, systemic inflammation, and comorbidity burden. Its clinical value is therefore mainly complementary, particularly when interpreted alongside NT-proBNP and imaging-derived functional or tissue phenotypes [36,38,84,103].

Redox biomarkers may likewise refine phenotyping and prognostic assessment. Lower free thiol concentrations have been associated with greater clinical severity, higher NT-proBNP values, and adverse outcomes, whereas malondialdehyde (MDA) and uric acid have shown associations with disease severity and prognosis in selected cohorts. The results support the concept that hemodynamic burden and systemic redox imbalance represent complementary biological dimensions of heart failure. However, heterogeneous populations, analytical variability, limited biological specificity, and the absence of validated thresholds currently preclude the routine isolated use of oxidative stress biomarkers for diagnosis or treatment guidance. At present, redox–fibrotic panels should be regarded primarily as complementary tools for risk stratification and biological phenotyping [25,27,32,116,117,118].

Biological phenotyping should be interpreted alongside imaging because circulating biomarkers alone cannot define the functional and tissue-level expression of cardiac remodeling. Echocardiographic indices of diastolic function, myocardial deformation, and RV–PA coupling characterize the functional consequences of remodeling, whereas CMR using LGE, native T1 mapping, and ECV provides complementary characterization of focal scar and diffuse interstitial remodeling. Accordingly, the combination of circulating biomarkers with echocardiographic and CMR phenotypes may support complementary heart failure phenotyping and risk stratification; however, prospective validation of an integrated redox–Galectin-3–imaging algorithm is still required [3,48,49,108].

Taken together, the strength of the available evidence differs substantially across the domains considered in this review. Evidence is strongest for the association between systemic redox imbalance and heart failure severity or adverse outcomes, supported by meta-analyses, multicenter cohorts, and consistent observational findings. Evidence for Galectin-3 as a complementary prognostic and phenotyping biomarker is moderate, but its interpretation is limited by renal function, systemic inflammation, comorbidity burden, and uncertain incremental value beyond established biomarkers. By contrast, integrated redox–Galectin-3–imaging algorithms remain exploratory because prospective studies simultaneously evaluating circulating biomarkers, echocardiography, cardiac magnetic resonance, and clinical outcomes are scarce [25,32,36,38,108,116].

The key clinical studies supporting the relevance of oxidative and fibrotic biomarkers, as well as of the multimarker approach, are summarized in Table 2.

**Figure 2:**
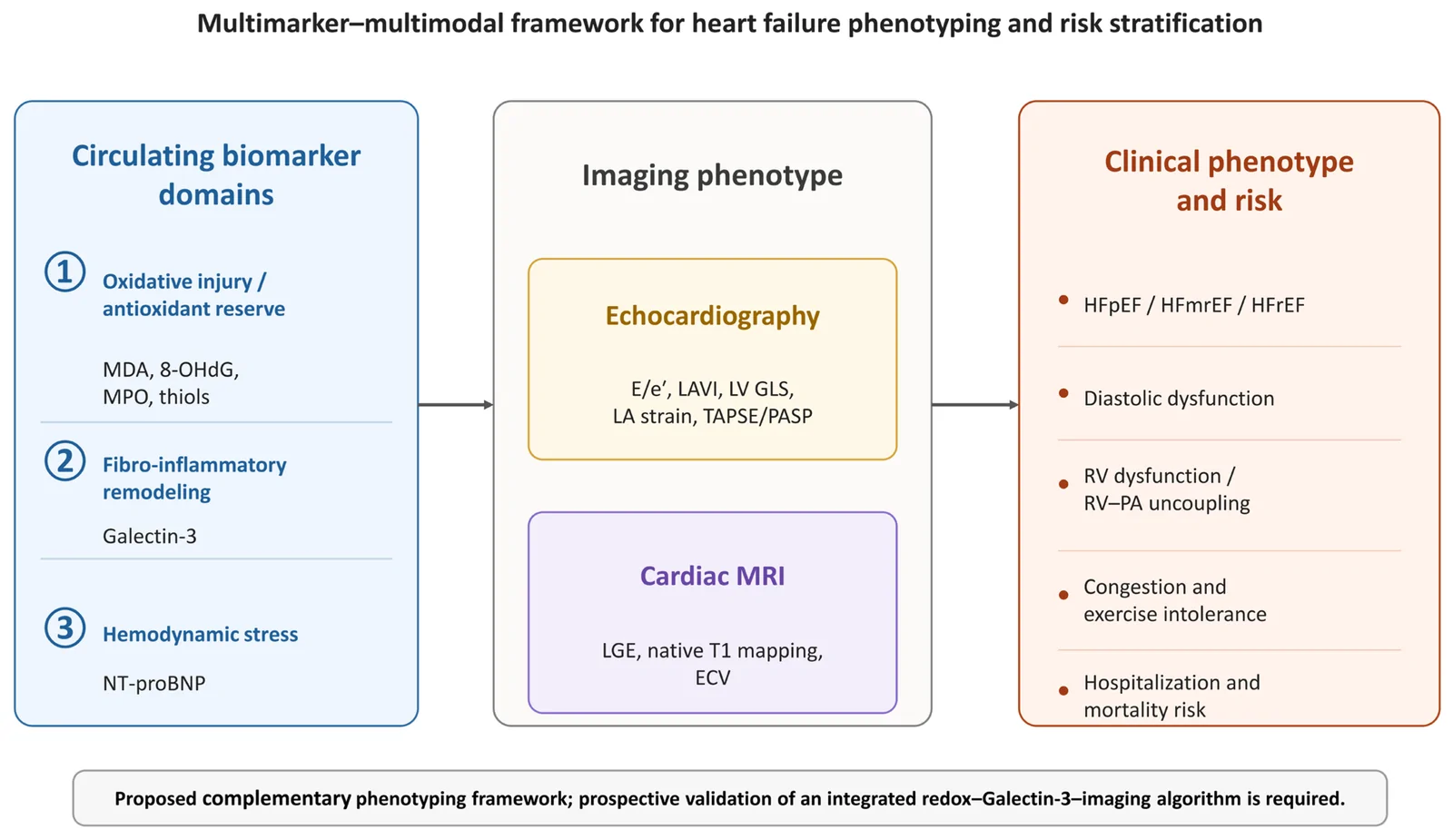
Proposed multimarker–multimodal framework for heart failure phenotyping and risk stratification. Circulating biomarkers reflecting oxidative injury, fibro-inflammatory remodeling, and hemodynamic stress may be interpreted alongside echocardiographic and cardiac magnetic resonance phenotypes to support complementary clinical phenotyping. Prospective validation of an integrated redox–Galectin-3–imaging algorithm is required. **Abbreviations**: MDA, malondialdehyde; 8-OHdG, 8-hydroxy-2′-deoxyguanosine; MPO, myeloperoxidase; NT-proBNP, N-terminal pro-B-type natriuretic peptide; E/e′, ratio of early mitral inflow velocity to early diastolic mitral annular velocity; LAVI, left atrial volume index; LV, left ventricular; GLS, global longitudinal strain; LA, left atrial; TAPSE, tricuspid annular plane systolic excursion; PASP, pulmonary artery systolic pressure; LGE, late gadolinium enhancement; ECV, extracellular volume fraction; MRI, magnetic resonance imaging; HFpEF, heart failure with preserved ejection fraction; HFmrEF, heart failure with mildly reduced ejection fraction; HFrEF, heart failure with reduced ejection fraction; RV, right ventricular; PA, pulmonary artery.

A proposed multimarker–multimodal framework merging circulating biomarkers with echocardiographic and CMR phenotypes is presented in Figure 2.

## 6. Expansion of Therapeutic Options and Precision Medicine

Standard therapies. Beyond their classical hemodynamic and neurohormonal effects, standard heart failure therapies may also be viewed through the lens of their influence on biological remodeling: angiotensin receptor–neprilysin inhibitors have been associated with reduced adverse remodeling, beta-blockers may indirectly reduce oxidative stress, and mineralocorticoid receptor antagonists are relevant because of their antifibrotic effects.

Emerging therapies. As shown by Ye et al. [119], Miao et al. [120], and Jie et al. [121], current interest is increasingly directed toward therapies capable of directly targeting the enzymatic sources of oxidative stress, especially NADPH oxidases. In parallel, Liu et al. [45] and Sabbah [122] point out the importance of mitochondria-targeted therapies, such as mitochondria-targeted ubiquinone (MitoQ) and elamipretide (SS-31), in the context of the central role of mitochondrial dysfunction in heart failure progression. In the same direction, Fang et al. [62], Huang et al. [123], and Xie et al. [63] support the relevance of ferroptosis inhibitors as interventions directed against cell death dependent on oxidative stress and iron, while Seropian et al. [39] and Wang et al. [93] support the idea that Galectin-3 inhibition could limit fibro-inflammatory remodeling and structural disease progression. These strategies are particularly attractive because they target the sources and mediators of redox-fibrotic remodeling, not only the final consequences of the disease.

Precision medicine. Within this framework, as suggested by Ng et al. [25] and Drăgoi et al. [117], multimarker phenotyping and therapy individualization represent natural directions for development. The integration of hemodynamic, oxidative, inflammatory, and fibrotic biomarkers may allow a more faithful biological characterization of the patient with heart failure, and in the future, combining these panels with advanced data analysis methods could support more precise models for therapeutic selection and for monitoring treatment response.

The major therapeutic targets discussed in the context of redox-dependent heart failure are presented in Table 3.

## 7. Therapeutic Implications and Future Perspectives

### 7.1. From Radical Scavenging to Targeting Redox Sources

Current evidence has shifted therapeutic interest from nonspecific antioxidant supplementation toward interventions targeting ROS-generating pathways and downstream remodeling processes. While initial strategies focused on the nonspecific neutralization of reactive oxygen species (ROS), contemporary data now support the need to intervene at the level of the intracellular sources of redox imbalance and the mediators that transform oxidative injury into irreversible structural remodeling.

### 7.2. The Lesson from the Past: Why Did Classical Antioxidants Fail?

The first therapeutic strategies directed against oxidative stress were built around the concept of nonspecific neutralization of reactive oxygen species. However, the clinical experience accumulated over the past decades has shown that this approach is insufficient to meaningfully modify the progression of heart failure. In this regard, the comprehensive review by D’Amato et al. [125] emphasizes that traditional antioxidant therapies, such as vitamins C and E, evaluated in multiple major clinical trials, have not demonstrated consistent benefits on cardiovascular mortality or disease progression. The authors explain this result through the mechanistic limitations of these agents: they act predominantly as scavengers, neutralizing already-formed ROS, without inhibiting the primary enzymatic sources of free radical production, such as NADPH oxidases or the mitochondrial respiratory chain, and without effectively penetrating critical intracellular compartments, especially the mitochondrial matrix.

This observation is important because, in heart failure, oxidative stress is not generated randomly, but arises from well-defined and persistent sources. Under conditions of chronic activation of NADPH oxidase 2 (NOX2) and NOX4 and altered mitochondrial function, as also highlighted in the experimental models discussed earlier, simple peripheral neutralization of radicals cannot interrupt the pathogenic circuit linking redox imbalance to inflammation, fibrosis, and structural remodeling. Thus, an essential conceptual shift has occurred: from the idea of “quenching” already-formed ROS to the need to directly block their sources of production and to limit the transformation of oxidative injury into irreversible tissue damage.

This transition is also supported by the systematic review by Jin and Kang [127], which analyzes both classical antioxidant supplements and newer pharmacologic and technological strategies. The authors show that although many antioxidant interventions have demonstrated favorable biochemical effects and, in certain contexts, some cardiovascular benefits, the overall clinical results have remained heterogeneous and dependent on the type of intervention, dose, disease context, and characteristics of the studied population. In parallel, the review highlights the growing interest in more targeted approaches directed toward ROS-generating sources and specific pathogenic circuits, including mitochondrial pathways, NADPH oxidase, xanthine oxidase, and nanoparticle-based targeted delivery systems. Thus, the authors indicate that simple nonspecific antioxidant supplementation is often insufficient and that future therapies must more precisely target the mechanisms generating and modulating oxidative stress.

The same direction is reflected in the recent review published by Singh [128] and colleagues, which argues for the need for a paradigm shift in cardiovascular redox therapy. The authors show that conventional antioxidants, such as vitamins C and E or β-carotene, although promising in experimental models, have not generated consistent cardiovascular clinical benefits in most human studies. Against this background, the review highlights the development of a new generation of antioxidant therapies directed toward specific pathogenic mechanisms, such as mitochondria-targeted antioxidants, NADPH oxidase inhibitors, Nrf2 pathway activators, and smart nanoparticle-based delivery systems. This work reinforces the idea that simple nonspecific neutralization of free radicals is not sufficient and that future therapeutic strategies must directly target the sources and biological networks sustaining oxidative stress.

Therefore, the major therapeutic implication of these data is that the treatment of heart failure can no longer be conceived exclusively in terms of reducing hemodynamic load or providing general antioxidant supplementation. Instead, future directions must aim at targeting the enzymatic and mitochondrial sources of ROS, protecting vulnerable intracellular compartments, and modulating the mediators that link oxidative stress to fibrosis and remodeling. In this new framework, redox therapy no longer means mere “neutralization,” but rather a mechanistically oriented intervention directed at the central links of disease progression [129].

### 7.3. Targeting the Enzymatic Sources of ROS: NADPH Oxidases

In the new therapeutic paradigm of heart failure, NADPH oxidases are important enzymatic sources of ROS in the myocardium and may contribute to redox-sensitive remodeling pathways. The data synthesized by Ye et al. [119], as well as the complementary reviews published by Miao et al. [120] and Jie et al. [121], support the view that the NOX2 and NOX4 isoforms are activated in the context of chronic hemodynamic and metabolic stress and directly contribute to the amplification of redox imbalance in the remodeling myocardium. Within this framework, NOX-derived ROS do not merely cause nonspecific oxidative injury, but also activate maladaptive signaling networks involved in disease progression. These include NF-κB, which sustains the inflammatory response, and the TGF-β/Smad axis, which is involved in fibroblast activation, myofibroblast differentiation, and progressive collagen accumulation. In parallel, other redox-sensitive pathways relevant to hypertrophy, apoptosis, and remodeling are also modulated, including MAPK, and in some contexts interactions with protein kinase B (Akt), mechanistic target of rapamycin (mTOR), and nuclear factor kappa B (NF-κB) signaling have also been described.

The pathogenic importance of NADPH oxidases derives precisely from this early position within the remodeling cascade. If ROS are neutralized only after they have already been generated, persistent enzymatic activation of NOX remains intact, and the inflammatory and profibrotic circuits continue to be stimulated. This explains, at least in part, why classical antioxidant strategies have had limited clinical results: they may reduce some of the peripheral oxidative stress, but they do not interrupt the enzymatic generator that sustains the pathological process. From this perspective, targeting NOX appears more rational than simply neutralizing already-formed radicals.

The review published by Miao et al. [120] reinforces this interpretation, showing that activation of NOX2 and NOX4 is associated with cardiomyocyte hypertrophy, apoptosis, interstitial fibrosis, and metabolic remodeling of the myocardium. The authors emphasize that these isoforms contribute to oxidative stress and participate in the structural and metabolic remodeling of the heart, making them attractive therapeutic targets. At the same time, the review by Jie et al. [121] extends the concept and shows that the relationship between NADPH oxidases and energy metabolism is bidirectional: NOX2 and NOX4 contribute to mitochondrial dysfunction and impaired energy homeostasis, and this bioenergetic disturbance in turn amplifies oxidative stress and cardiac remodeling. These observations suggest a reciprocal relationship between NOX activation, mitochondrial dysfunction, and adverse remodeling.

However, these data also require a certain degree of caution. Especially in the case of NOX4, the recent literature suggests a complex, and at times even partially compensatory, role depending on the cellular context and stage of disease. Therefore, the development of selective NOX2 and NOX4 inhibitors remains a promising direction, but requires further validation before it can be broadly translated into clinical practice. Overall, however, the current data clearly support the idea that future therapies should target the enzymatic sources of oxidative stress, not only its final products, and NADPH oxidases are among the most relevant targets in this regard.

### 7.4. The Mitochondrion as a Major Therapeutic Target

In parallel with the growing interest in inhibiting enzymatic ROS sources, mitochondria are being investigated as therapeutic targets because of their central role at the intersection of energy metabolism, oxidative stress, and cell death. Liu et al. [45] frame mitochondrial dysfunction as a fundamental mechanism of disease progression, emphasizing that the mitochondrion serves as both the site of an energy deficit characterized by reduced ATP production and a major source of mitochondria-derived reactive oxygen species. This excess mtROS has direct consequences for the cardiomyocyte, through activation of apoptotic pathways, disruption of Ca^2+^ homeostasis, and promotion of maladaptive remodeling.

Mitochondrial Ca^2+^ overload and respiratory-chain dysfunction may create a self-amplifying cycle of membrane-potential loss, reduced ATP production, mtROS generation, and cardiomyocyte death. Elamipretide (SS-31/MTP-131) is being investigated as a potential intervention to interrupt this cycle by preserving cardiolipin integrity and mitochondrial function [45,122].

Mitochondria-targeted therapies such as MitoQ and elamipretide (SS-31/MTP-131) have shown preclinical and translational potential to improve mitochondrial respiration, reduce ROS production, preserve ATP synthesis, and attenuate ventricular remodeling. However, the available evidence remains predominantly experimental, and no mitochondria-directed strategy has yet demonstrated sufficient clinical efficacy for routine use in heart failure. Thus, the mitochondrion should be regarded as a central therapeutic target, but one requiring further translational validation [45,122].

Thus, protection of mitochondrial function, limitation of mtROS production, stabilization of Ca^2+^ homeostasis, and prevention of pathological mPTP opening emerge as essential directions for future therapies capable of interrupting the vicious circle between redox imbalance, energetic collapse, and progressive structural remodeling.

### 7.5. SGLT2 Inhibitors—Moving from Redox Mechanisms to Clinical Benefit

In this context, SGLT2 inhibitors (dapagliflozin, empagliflozin) represent the first clinical example of a therapy with indirectly demonstrated redox impact [130,131].

According to the analysis by Fender and Dobrev [124], SGLT2 inhibitors exert cardioprotective effects through pleiotropic mechanisms that include:

#### 7.5.1. Ionic Modulation (NHE1)

SGLT2 inhibitors lead to a reduction in intracellular Na^+^, probably through interference with the sodium-hydrogen exchanger 1 (NHE1). This reduction in cytosolic Na^+^ limits Ca^2+^ overload (via the sodium–calcium exchanger (NCX)), thereby improving mitochondrial function and reducing ROS production. Fender and Dobrev emphasize that these effects are independent of glycemic control and contribute to normalization of cellular homeostasis.

#### 7.5.2. Modulation of Mitochondrial Function

SGLT2 inhibitors are frequently associated with reduced oxidative stress markers in experimental models and with improved mitochondrial function. One of the mechanisms through which they act is the activation of AMP-activated protein kinase (AMPK) and the sirtuins sirtuin 1 (SIRT1) and sirtuin 3 (SIRT3), nicotinamide adenine dinucleotide (NAD^+^)-dependent enzymes that regulate energy metabolism and the antioxidant response.

SIRT1 (predominantly nuclear) modulates inflammation and metabolic adaptation, whereas SIRT3 (mitochondrial) activates antioxidant enzymes such as superoxide dismutase 2 (SOD2) and optimizes respiratory chain function. Through these pathways, autophagy and mitophagy are stimulated, which are processes involved in the recycling and elimination of dysfunctional mitochondria, thereby limiting the accumulation of damaged organelles and reducing mitochondrial reactive oxygen species production.

#### 7.5.3. Inhibition of the NLRP3 Inflammasome

Fender and Dobrev describe reduced activation of NLRP3, caspase-1, and IL-1β under SGLT2 inhibitor treatment, suggesting limitation of the transition from inflammation to fibrosis.

According to D’Amato et al. [125] and Ye et al. [119], SGLT2 inhibitors may also reduce activation of NADPH oxidases, indicating a convergence between mitochondrial protection and reduction in enzymatic oxidative stress. However, Liu et al. [45] emphasize that some mitochondrial mechanisms remain hypothetical and require further validation.

Thus, in conclusion, SGLT2 inhibitors are not “classical antioxidants,” but agents with complex metabolic actions that reduce oxidative stress by influencing ROS-generating mechanisms at their source, rather than by simply neutralizing radicals that have already been formed [99,124,130,131].

### 7.6. Targeting Galectin-3: A Preclinical Antifibrotic Strategy

Galectin-3 is a candidate downstream target because experimental studies link it to macrophage activation, fibroblast signaling, and extracellular matrix deposition. In experimental models, genetic deletion or pharmacological inhibition of Galectin-3 attenuated oxidative stress, fibrosis, adverse remodeling, and ventricular dysfunction. The results are consistent with an active contribution of Galectin-3 to chronic fibro-inflammatory remodeling in experimental models [39,92].

In post-infarction experimental models, pharmacological inhibition with the Galectin-3 C-terminal domain (Gal-3C) reduced interstitial fibrosis, scar expansion, ventricular dilation, and inflammatory–profibrotic signaling.

More broadly, direct antifibrotic approaches remain limited by heterogeneity across disease etiologies, stages, and myocardial regions, as well as by the absence of reliable fibrosis-specific biomarkers for patient stratification and therapeutic monitoring [132]. Nevertheless, evidence for Galectin-3 inhibition remains predominantly preclinical. No Galectin-3-directed therapy has yet demonstrated a clinical therapeutic benefit in heart failure, and its potential role should therefore be regarded as a mechanistically targeted antifibrotic strategy requiring further translational validation [93,126].

### 7.7. Integrating Redox Biomarkers into Precision Medicine

The translation of redox biology into precision-oriented heart failure care requires an integrated rather than single-marker approach. Biomarkers of oxidative injury, including MDA, 8-OHdG, MPO, and uric acid, may provide information complementary to measures of antioxidant reserve such as circulating thiols and to fibro-inflammatory biomarkers such as Galectin-3. Available clinical studies associate several of these markers with disease severity and adverse outcomes; however, heterogeneity in assays, biological samples, patient populations, and outcome definitions currently prevents the establishment of standardized redox thresholds or routine treatment-guiding algorithms [25,116,118].

A proposed redox–fibrotic phenotype could therefore integrate oxidative injury, antioxidant reserve, fibro-inflammatory remodeling, NT-proBNP, and imaging-derived functional and tissue characteristics. Such an approach may improve biological phenotyping, risk stratification, and the identification of patients most likely to benefit from mechanism-oriented therapies. Nevertheless, this construct remains hypothesis-generating and requires prospective validation before it can be incorporated into clinical decision-making [3,108,117].

## 8. Limitations of Present Evidence

The interpretation of redox and fibro-inflammatory biomarkers in heart failure is limited by substantial heterogeneity in the available literature. Studies have evaluated diverse markers—including MDA, 8-epi-prostaglandin F2α (8-epi-PGF2α), OSI/TOS, thiols, MPO, xanthine oxidoreductase (XOR), and uric acid—across heterogeneous populations and heart failure phenotypes. The apparent inconsistencies among studies are likely explained by differences in disease stage, ischemic versus non-ischemic etiology, renal function, comorbidity burden, biological sample, assay methodology, timing of biomarker collection, study design, and outcome definition. Consequently, findings obtained for MDA, thiols, Galectin-3, and other redox-related markers should not be generalized across all heart failure populations, and neither universal thresholds nor a standardized minimal redox–fibrotic biomarker panel can currently be recommended [25,116,117,118].

Methodological limitations are particularly relevant for commonly used markers such as MDA and circulating thiols. MDA may be assessed by thiobarbituric acid reactive substances (TBARS)-based spectrophotometry, enzyme-linked immunosorbent assay (ELISA), or chromatographic techniques, each with different specificity, sensitivity, and susceptibility to pre-analytical conditions. In addition, circulating redox biomarkers may be influenced by renal dysfunction, diabetes, hypertension, dyslipidemia, systemic inflammation, and concomitant therapies. These factors complicate separation of heart-failure-specific oxidative injury from broader systemic redox disturbances and limit the transferability of proposed biomarker thresholds [25,116,118].

Most clinical evidence remains observational and therefore does not establish causal relationships between circulating redox markers, myocardial remodeling, and clinical outcomes. Similarly, many mechanistic concepts—including mitochondrial ROS generation, altered fusion–fission dynamics, ferroptosis, and RV bioenergetic failure—derive predominantly from cellular and animal models. Although these studies provide biological plausibility, direct extrapolation to human heart failure requires caution, particularly when translating experimental therapeutic targets into clinical interventions [40,45,98,100,116,127].

Future studies should use standardized pre-analytical and analytical procedures, prespecified biomarker panels, phenotype-specific patient stratification, and longitudinal assessment alongside echocardiography, CMR, and hard clinical outcomes. Only prospective validation in adequately powered cohorts can determine whether integrated redox–Galectin-3–imaging phenotypes improve prediction beyond established clinical models and natriuretic peptides, and whether they can ultimately guide treatment selection. Until then, redox–fibrotic biomarker panels should be regarded as complementary research and risk-phenotyping tools rather than routine clinical decision algorithms [3,113,117].

## 9. Conclusions

HF is not solely a hemodynamic disorder. Oxidative stress, mitochondrial dysfunction, inflammation, and interstitial fibrosis interact throughout disease progression.

The analyses by D’Amato et al. [125], Jin and Kang [127], and Singh et al. [128] show that simple nonspecific neutralization of free radicals is insufficient to modify disease progression, thereby supporting the need for a therapeutic paradigm shift directed toward the sources and biological circuits of oxidative stress. In this regard, Ye et al. [119], Miao et al. [120], and Jie et al. [121] highlight the central role of NADPH oxidases, especially the NOX2 and NOX4 isoforms, in initiating and sustaining maladaptive inflammatory, profibrotic, and metabolic cascades.

In turn, Liu et al. [45] and Sabbah [122] show that the mitochondrion represents not only the site of bioenergetic collapse, but also a major source of mtROS and an essential therapeutic target, particularly through interventions capable of limiting Ca^2+^ overload, mPTP opening, and the loss of oxidative phosphorylation efficiency. Downstream of oxidative injury, Seropian et al. [39], Vlachou et al. [92], Wang et al. [93], and Bouffette et al. [126] indicate that Galectin-3 goes beyond the status of a simple biomarker and acts as an active mediator of fibro-inflammatory remodeling, representing a promising therapeutic target, although clinical validation remains limited.

Finally, the data synthesized by Chawla et al. [116], Ng et al. [25], and Drăgoi et al. [117] outline the perspective of precision medicine, suggesting that the integration of redox biomarkers, antioxidant reserve markers, and fibro-inflammatory biomarkers could allow the definition of multimarker signatures capable of refining risk stratification and personalizing therapeutic intervention.

Future therapies may need to target both hemodynamic burden and ROS-generating pathways, mitochondrial dysfunction, inflammation, and fibrosis. Clinically, this framework supports the integration of redox biomarkers and Galectin-3 with NT-proBNP, functional echocardiographic indices, and CMR markers of focal and diffuse interstitial remodeling. Such multimarker–multimodal phenotyping may improve risk stratification and shape future precision-oriented management; however, no validated clinical algorithm currently integrates these domains, and prospective studies are required [3,4,48,49,108].

## Figures and Tables

**Table 1:** Classification of biomarkers in heart failure.

Category	Biomarkers	Pathophysiology Reflected	Clinical Role	Ref.
**Hemodynamic**	NT-proBNP	Myocardial wall stress, hemodynamic overload, congestion	Central biomarker for diagnosis, assessment of disease severity, and risk stratification	[1,2,3]
**Oxidative**	MDA, 8-OHdG, MPO, uric acid	Lipid peroxidation, oxidative DNA damage, oxidative-inflammatory activation, systemic redox imbalance	Complementary biomarkers for prognosis, assessment of biological disease severity, and redox status phenotyping	[3,12,18,19,20,21,22,23,24]
**Antioxidant/redox reserve**	Free thiols, total/native thiols, TAC/TAS, catalase, ceruloplasmin	Systemic antioxidant reserve, compensatory capacity against ROS, redox homeostasis	Complementary biomarkers of antioxidant defense depletion and disease severity	[29,30,31,32,33,34]
**Fibrotic/remodeling**	Galectin-3	Inflammation, macrophage activation, interstitial fibrosis, structural remodeling	Complementary biomarker for prognosis and phenotyping, especially relevant in HFpEF and in multimarker strategies	[19,35,36,37,38,39]

**Abbreviations**: NT-proBNP, N-terminal pro-B-type natriuretic peptide; MDA, malondialdehyde; 8-OHdG, 8-hydroxy-2′-deoxyguanosine; MPO, myeloperoxidase; TAC, total antioxidant capacity; TAS, total antioxidant status; ROS, reactive oxygen species; HFpEF, heart failure with preserved ejection fraction; Gal-3, Galectin-3.

**Table 2:** Key clinical and translational studies informing oxidative, fibrotic, and multimarker phenotyping in heart failure.

Study	Population	Biomarker	Main Finding	Ref.
BIOSTAT-CHF	Patients with new-onset or worsening heart failure	Free thiols	Lower free thiol levels were associated with more advanced NYHA class, higher NT-proBNP values, and increased risk of mortality and adverse events	[32]
Romuk et al.	Patients with chronic heart failure	MDA, uric acid	MDA and uric acid correlated with disease severity and showed prognostic value for adverse outcomes	[27]
Gtif et al.	Patients with HFrEF	TAC, uric acid + NT-proBNP	The prognostic model integrating oxidative markers together with NT-proBNP showed better predictive performance than conventional assessment	[29]
Shi et al.	Meta-analysis in patients with HFpEF	Galectin-3	Galectin-3 was associated with the risk of de novo HFpEF, unfavorable prognosis, and severity of diastolic dysfunction	[38]
DIAST-CHF	Patients with cardiovascular risk factors evaluated for HFpEF	Galectin-3	Galectin-3 showed moderate diagnostic utility and was associated with incident HFpEF, mortality, and adverse events	[84]
WISE-CVD	Women with ischemia and no obstructive coronary artery disease	Cystine	Elevated cystine levels were associated with higher end-diastolic pressures and impaired ventricular filling, suggesting a link between oxidative stress and early diastolic dysfunction	[94]
CECCY	Patients exposed to anthracyclines	MPO, Galectin-3	Chemotherapy was associated with increased MPO and Galectin-3; elevated baseline MPO was associated with more pronounced subclinical myocardial injury, whereas Galectin-3 was not a robust predictor of acute ventricular dysfunction	[104]
EMPEROR-Reduced/Pocock et al.	Patients with HFrEF	NT-proBNP, hs-cTnT	Integration of NT-proBNP and hs-cTnT into a simple model enabled robust and easily applicable prognostic stratification	[115]
Framingham Heart Study	General population cohort	Galectin-3 (serial)	An increase in Galectin-3 over time was associated with a higher risk of incident heart failure, cardiovascular disease, and mortality	[89]

**Abbreviations**: BIOSTAT-CHF, A Systems BIOlogy Study to TAilored Treatment in Chronic Heart Failure; CECCY, Carvedilol Effect in Preventing Chemotherapy-Induced Cardiotoxicity; DIAST-CHF, Diastolic Dysfunction in Heart Failure; EMPEROR-Reduced, Empagliflozin Outcome Trial in Patients with Chronic Heart Failure and a Reduced Ejection Fraction; HFrEF, heart failure with reduced ejection fraction; HFpEF, heart failure with preserved ejection fraction; hs-cTnT, high-sensitivity cardiac troponin T; MDA, malondialdehyde; MPO, myeloperoxidase; NT-proBNP, N-terminal pro-B-type natriuretic peptide; NYHA, New York Heart Association; TAC, total antioxidant capacity; WISE-CVD, Women’s Ischemia Syndrome Evaluation–Coronary Vascular Dysfunction.

**Table 3:** Major therapeutic targets in redox-dependent heart failure.

Target	Therapy/Strategy	Mechanism	Evidence	Ref.
SGLT2-related metabolic/redox modulation	Dapagliflozin, empagliflozin	Reduce intracellular Na^+^ and Ca^2+^ overload, attenuate mitochondrial dysfunction, reduce ROS production, activate AMPK/SIRT1/SIRT3, and limit NLRP3 activation	Clinical + mechanistic/indirect redox evidence	[52,119,124,125]
NADPH oxidases (NOX2/NOX4)	NOX targeting/NOX inhibition	Target major enzymatic sources of ROS and may limit activation of inflammatory and profibrotic pathways	Mechanistic/preclinical/review-supported	[46,119,120,121]
Mitochondria	MitoQ, SS-31 (elamipretide)	Reduce mtROS, stabilize mitochondrial function, limit pathological opening of the mPTP, and improve cellular bioenergetics	Preclinical/translational	[45,122]
Ferroptosis pathway	Ferrostatin-1, iron chelation, anti-ferroptotic strategies	Limit iron-dependent lipid peroxidation and reduce oxidative stress-associated cardiomyocyte death	Preclinical/mechanistic	[62,63,123]
Galectin-3 axis	Galectin-3 inhibition (e.g., Gal-3C)	Reduce interstitial fibrosis and fibro-inflammatory remodeling	Preclinical/experimental	[39,92,93,126]
Classical antioxidant therapy	Non-specific antioxidant supplementation	Non-specific neutralization of already formed ROS	Negative/inconsistent clinical evidence	[125,127,128]

**Abbreviations**: AMPK, AMP-activated protein kinase; Ca^2+^, calcium ion; Gal-3, Galectin-3; Gal-3C, Galectin-3C; MitoQ, mitochondria-targeted ubiquinone/mitoquinone; mPTP, mitochondrial permeability transition pore; mtROS, mitochondrial reactive oxygen species; Na^+^, sodium ion; NADPH, nicotinamide adenine dinucleotide phosphate; NLRP3, NOD-, LRR- and pyrin domain-containing protein 3 inflammasome; NOX, NADPH oxidase; NOX2/NOX4, NADPH oxidase isoforms 2 and 4; ROS, reactive oxygen species; SGLT2, sodium–glucose cotransporter 2; SIRT1/SIRT3, sirtuin 1/sirtuin 3; SS-31, elamipretide.

## Data Availability

No new data were created or analyzed in this study. Data sharing is not applicable to this article.

## Abbreviations

The following abbreviations are used in this manuscript:

3-NT	3-nitrotyrosine
4-HNE	4-hydroxynonenal
8-epi-PGF2α	8-epi-prostaglandin F2α
8-OHdG	8-hydroxy-2′-deoxyguanosine
ACSL4	acyl-CoA synthetase long-chain family member 4
AGE/AGEs	advanced glycation end product(s)
Akt	protein kinase B
ALDH2	aldehyde dehydrogenase 2
AMI	acute myocardial infarction
AMPK	AMP-activated protein kinase
ARIC	Atherosclerosis Risk in Communities
ATP	adenosine triphosphate
BAK	Bcl-2 homologous antagonist/killer
BAP	biological antioxidant potential
BAX	Bcl-2-associated X protein
BIOSTAT-CHF	A Systems BIOlogy Study to TAilored Treatment in Chronic Heart Failure
BNP	B-type natriuretic peptide
CA-125	carbohydrate antigen 125
Ca^2+^	calcium ion
cGAS-STING	cyclic GMP–AMP synthase–stimulator of interferon genes
cGMP-PKG	cyclic guanosine monophosphate–protein kinase G
CECCY	Carvedilol Effect in Preventing Chemotherapy-Induced Cardiotoxicity
CMR	cardiac magnetic resonance
COPD	chronic obstructive pulmonary disease
CT-1	cardiotrophin-1
DAMP	damage-associated molecular pattern
DIAST-CHF	Diastolic Dysfunction in Heart Failure
DNA	deoxyribonucleic acid
Drp1	dynamin-related protein 1
dROMs	derivatives of reactive oxygen metabolites
E/A	ratio of early to late transmitral flow velocities
E/e′	ratio of early mitral inflow velocity to early diastolic mitral annular velocity
ECV	extracellular volume fraction
ELISA	enzyme-linked immunosorbent assay
EMPEROR-Reduced	Empagliflozin Outcome Trial in Patients with Chronic Heart Failure and a Reduced Ejection Fraction
eNOS	endothelial nitric oxide synthase
ERK1/2	extracellular signal-regulated kinase 1/2
ETC	electron transport chain
F2-isoprostanes	F2-isoprostanes
Gal-3	galectin-3
Gal-3C	galectin-3 C-terminal domain
GDF-15	growth differentiation factor 15
GLM	generalized linear model
GLS	global longitudinal strain
GPX4	glutathione peroxidase 4
GSH	glutathione
HF	heart failure
HFmrEF	heart failure with mildly reduced ejection fraction
HFpEF	heart failure with preserved ejection fraction
HFrEF	heart failure with reduced ejection fraction
HIF-1α	hypoxia-inducible factor 1-alpha
Hmox1	heme oxygenase 1
HPLC	high-performance liquid chromatography
hs-cTn	high-sensitivity cardiac troponin
hs-cTnT	high-sensitivity cardiac troponin T
hs-CRP	high-sensitivity C-reactive protein
IFN-β	interferon beta
IKKβ	inhibitor of nuclear factor kappa-B kinase subunit beta
IL-1β	interleukin-1 beta
INOCA	ischemia with no obstructive coronary artery disease
iNOS	inducible nitric oxide synthase
IRF3	interferon regulatory factor 3
JNK	c-Jun N-terminal kinase
LA	left atrial
LAVI	left atrial volume index
LGE	late gadolinium enhancement
LV	left ventricular
LVEDP	left ventricular end-diastolic pressure
LVEF	left ventricular ejection fraction
LVMI	left ventricular mass index
M2	type 2/alternatively activated macrophage phenotype
MAPK	mitogen-activated protein kinase
MDA	malondialdehyde
MFF	mitochondrial fission factor
Mfn1/2	mitofusin 1/2
mGPX4	mitochondrial glutathione peroxidase 4
MPI	myocardial performance index
MitoQ	mitochondria-targeted ubiquinone/mitoquinone
MPO	myeloperoxidase
MPRI	myocardial perfusion reserve index
mPTP	mitochondrial permeability transition pore
mtDNA	mitochondrial DNA
mTOR	mechanistic target of rapamycin
MTP-131	mitochondria-targeted peptide 131/elamipretide
mtROS	mitochondria-derived reactive oxygen species
Na^+^	sodium ion
NAD^+^	nicotinamide adenine dinucleotide
NADPH	nicotinamide adenine dinucleotide phosphate
NCX	sodium–calcium exchanger
NF-κB	nuclear factor kappa B
NHE1	sodium–hydrogen exchanger 1
NLRP3	NOD-like receptor family pyrin domain containing 3
NO	nitric oxide
NOX	NADPH oxidase
NOX2	NADPH oxidase 2
NOX4	NADPH oxidase 4
Nrf2	nuclear factor erythroid 2-related factor 2
NT-proBNP	N-terminal pro-B-type natriuretic peptide
NYHA	New York Heart Association
OPA1	optic atrophy 1
OSI	oxidative stress index
PA	pulmonary artery
p38-MAPK	p38 mitogen-activated protein kinase
PASP	pulmonary artery systolic pressure
PKC	protein kinase C
PSH	protein sulfhydryl groups
PUFA	polyunsaturated fatty acid
RAGE	receptor for advanced glycation end products
RIRR	ROS-induced ROS release
ROC	receiver operating characteristic
ROS	reactive oxygen species
RV	right ventricle
RV–PA	right ventricular–pulmonary arterial
RyR2	ryanodine receptor 2
s′	tissue Doppler systolic annular velocity
SGLT2	sodium–glucose cotransporter 2
SHp	serum thiol groups
SIRT1	sirtuin 1
SIRT3	sirtuin 3
SLC7A11	solute carrier family 7 member 11
Smad2/3	small mothers against decapentaplegic 2/3
SOD2	superoxide dismutase 2
SS-31	elamipretide
sST2	soluble suppression of tumorigenicity 2
System xc−	cystine/glutamate antiporter system xc−
TAC	total antioxidant capacity
TAPSE	tricuspid annular plane systolic excursion
TAS	total antioxidant status
TBARS	thiobarbituric acid reactive substances
TBK1	TANK-binding kinase 1
TGF-β	transforming growth factor beta
TOS	total oxidant status
VDAC	voltage-dependent anion channel
VDAC1	voltage-dependent anion channel 1
VO_2_max	maximal oxygen uptake
WISE-CVD	Women’s Ischemia Syndrome Evaluation–Coronary Vascular Dysfunction
XOR	xanthine oxidoreductase
